# Prognostic and clinicopathological value of GATA binding protein 3 in breast cancer: A systematic review and meta-analysis

**DOI:** 10.1371/journal.pone.0174843

**Published:** 2017-04-10

**Authors:** Yawen Guo, Pan Yu, Zeming Liu, Yusufu Maimaiti, Chen Chen, Yunke Zhang, Xingjie Yin, Shan Wang, Chunping Liu, Tao Huang

**Affiliations:** 1 Department of Breast and Thyroid Surgery, Union Hospital, Tongji Medical College, Huazhong University of Science And Technology, Wuhan, China; 2 Department of General Surgery, People’s Hospital of Xinjiang Uygur Autonomous Region, Urumqi, China; Fondazione IRCCS Istituto Nazionale dei Tumori, ITALY

## Abstract

The potential prognostic value of GATA binding protein 3 (GATA3) in breast cancer has recently increased, although the evidence is inconclusive. This meta-analysis of 10 articles involving 5,080 breast cancer patients explored the prognostic and clinicopathological value of GATA3 in breast cancer. Time to tumor progression (TTP) and overall survival (OS) were primary endpoints. Pooled hazard ratio (HR), pooled risk ratio (RR), and 95% confidence interval (CI) were calculated to evaluate the association between GATA3, prognosis, and clinicopathological parameters. High GATA3 expression predicts breast cancer, with a HR (HR = 0.671; 95% CI = 0.475–0.947; P = 0.023) of TTP, but is not associated with OS (HR = 0.889; 95% CI = 0.789–1.001; P = 0.052). GATA3 overexpression is associated with positive ER (RR = 3.155; 95% CI = 1.680–5.923; P = 0.000), positive PR (RR = 3.949; 95% CI = 1.567–9.954, P = 0.004), lower nuclear grade (RR = 0.435; 95% CI = 0.369–0.514; P = 0.000), and smaller tumor size (RR = 0.816; 95% CI = 0.709–0.940; P = 0.005). High GATA3 expression may predict TTP in breast cancer, and such patients may show better clinicopathological features.

## Introduction

Transcription factors or trans-acting factors are often organized in multigene families and play essential roles in activating target genes of specific cell fates by binding to their cognate DNA sequence to aid (and sometimes inhibit) RNA polymerase II (pol II) in locating the proper initiation site for transcription [1, 2]. The GATA family of transcription factors, which is composed of six highly conserved transcription factors, binds a consensus DNA sequence (A/T)GATA(A/G) in the promoters of target genes via two zinc-finger domains with the consensus sequence CX2CX17CX2C to directly activate or repress target gene expression [3].

GATA transcription factors play a wide role in determination of cell differentiation and control of cell proliferation and movement. GATA1, GATA2, and GATA3 are expressed primarily in hematopoietic cells and are linked to their specification, while GATA4, GATA5, and GATA6 play key roles in the specification of mesoderm and endoderm-derived tissues including the heart, intestines, and lungs [4]. In particular, GATA3 is also present in non-hematopoietic tissues including the kidneys [5], central nervous system [6], endothelial cells [7], and mammary gland [8], regulating their specification and differentiation.

During puberty, pregnancy, lactation, and involution in women, the mammary gland undergoes morphologic changes including cellular proliferation, differentiation, and apoptosis [9]. GATA3 plays a vital role in orchestrating the lineage determination and maturation of these cells by directing mammogenesis towards a luminal cell fate [10]. Even though GATA3 is so important to the mammary gland, how GATA3 influences survival of breast carcinoma patients remains an important question. To our knowledge, no meta-analyses have been performed on this topic thus far. Here, we performed a meta-analysis to explore determine the prognostic and clinicopathological value of GATA3 in breast cancer.

## Materials and methods

### Data sources and keywords

We searched studies without language restrictions using the bibliographic databases PubMed/MEDLINE, Web of Science, EMBASE, and the Cochrane Library databases from inception to July 29, 2016. Both Medical Subject Headings (MeSH) terms and free-text words were used in the search strategy to increase sensitivity. The key terms were “GATA3,” “HDR,” “HDRS,” “GATA binding protein 3,”; “breast cancer,” “breast carcinoma,” or “breast tumor”; and “survival,” “outcome,” or “prognosis.” Additional relevant papers were obtained through extensive crosschecking of the reference lists of all retrieved articles, if necessary.

### Inclusion and exclusion criteria

Studies were selected for meta-analysis if they fulfilled the following criteria: (1) studies must have focused on breast cancer; (2) all patients must have had breast cancer diagnoses confirmed via pathological examination; (3) the expression level of GATA3 protein must be analyzed in tumor tissues by immunohistochemical staining (IHC); (4) adequate data of pooled hazard ratios (HRs) and 95% confidence intervals (CIs); and (5) the correlation between GATA3 and clinicopathological features was discussed. Exclusion criteria were as follows: (1) duplicate publication; (2) non-English papers where non-human experiments were performed; (3) conference abstract; (4) letters, reviews, case reports, editorials, and comments; (5) not a prognostic study about breast cancer; (6) insufficient data regarding 95% CI and HRs provided, and a Kaplan-Meier curve could not be extracted. In addition, for studies where more than one article was published from a single center, the article with the information most relevant to this study was included [11].

### Data extraction

Data extraction was conducted independently by two investigators (Yawen Guo and Pan Yu) from selected studies according to the Preferred Reporting Items for Systematic Reviews and Meta-Analyses (PRISMA) statement [12]. For each relevant parameter, data were extracted including the following: surname of first author, year of publication, country, number of patients, specimen, detection method for GATA3 protein expression, cut-off value for the rates of GATA3 positive/high expression, duration of follow-up after surgery, and end-point. To evaluate the quality of the studies included we used the Newcastle-Ottawa Scale (NOS) for each study, and a score ≥ 6 was considered a high quality study [13]. Disagreement between investigators was resolved by consensus. Engauge Digitizer software, version 4.1 (http://digitizer.sourceforge.net) was utilized to reconstruct the HRs estimate and its variance. For articles that only provided survival data in a Kaplan Meier curve, we assumed that patients were censored at a constant rate during follow-up.

### Statistical analyses

Statistical analysis was performed according to the guidelines proposed by the MetaAnalysis of Observational Studies in Epidemiology (MOOSE) group [14]. Heterogeneity analysis was carried out by calculating the I^2^, which was interpreted as low (25%), moderate (50%), or high (75%); a P value of > 0.10 indicated a lack of heterogeneity among studies [12]. Pooled HRs and 95% CIs were obtained for the two outcome endpoints overall survival (OS) and time to tumor progression (TTP), which is a unified prognostic parameter combining disease-free survival (DFS) and relapse-free survival (RFS), cause they all means that the disease progression, via a fixed effects model if there was an absence of statistical heterogeneity among the studies. A random-effects model was adopted if heterogeneity was observed. An HR > 1 indicated worse survival for patients with high GATA3 expression, whereas an HR < 1 implied a survival benefit. What is more, pooled risk ratios (RRs) and their 95% CIs were used to assess the correlations between GATA3 expression and several clinicopathological features of breast cancer including the expression of ER, PR, Her-2, and Ki67; tumor size; lymph node metastasis; and nuclear grade. Forest plots were sorted according to the name of the first author, year, and country to illustrate HRs and RRs. Begg’s funnel plots and the Egger’s test were employed to estimate possible publication bias. We also performed sensitivity analysis by omitting each study or specific studies to find potential outliers. All these statistical analyses were performed using Stata version 12.0 (StataCorp, College Station, Texas, USA). P values for all comparisons were two-tailed and statistical significance was defined as P < 0.05.

## Results

### Study characteristics

**Fig 1:**
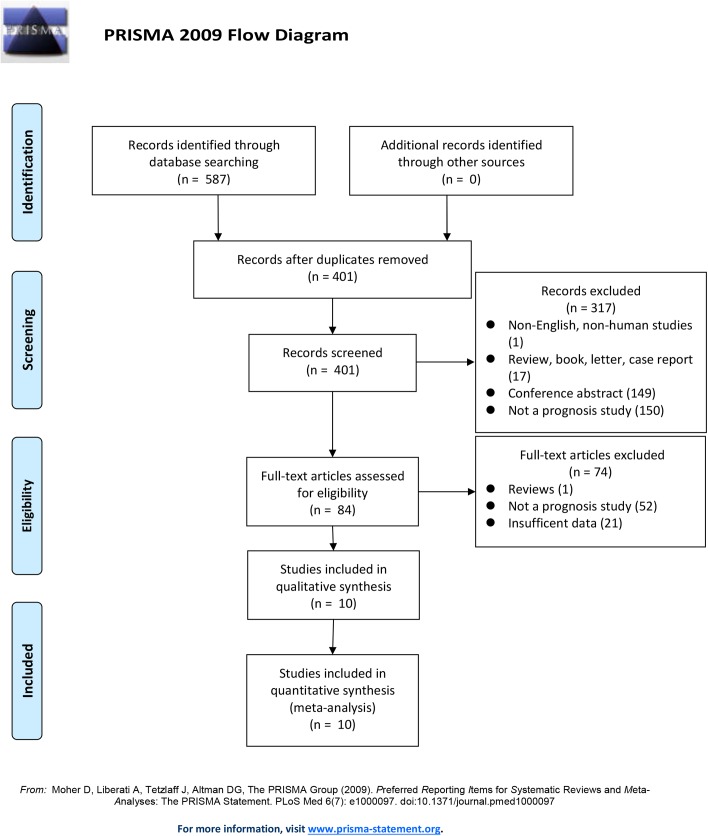
Flow chart of study selection.

A total of 587 articles were identified after the literature search from PubMed/MEDLINE, Web of Science, EMBASE, and the Cochrane Library databases utilizing different combinations of key terms. After reviewing titles and abstracts, 317 articles were excluded for being a non-English, non-human study (n = 1); a review, book, letter, or case report (n = 17); a conference abstract (n = 149); or not a prognostic study (n = 150). Additionally, 74 articles were excluded after screening the complete text because they were reviews (n = 1), not a prognostic study (n = 52), or had insufficient data (n = 21). The remaining 10 articles involving 5080 patients fulfilled our inclusion criteria [15–24]. The process of study selection is shown as a flow diagram (Fig 1). In addition, after evaluating the quality of all selected articles with the Newcastle-Ottawa Scale (NOS), each showed a high score (Table 1).

**Table 1:** Basic characteristics of the studies included in this meta-analysis evaluating survival in breast cancer patients.

Author	Year	Country	Number of patients	Specimen	Detection method	Cut-off (positive/high expression)	Follow up (months)	End-point	NOS score
Mehra	2005	America	139	Tissue	IHC	a median intensity of > 2.5	106.8 (1.5–204)	DFS/OS	7
Voduc	2008	Canada	3119	Tissue	IHC	> 5% positive nuclear staining	151.2 (1–222)	BCSS/RFS/DFS/DRFS/OS	8
Albergaria	2009	Portugal	249	Tissue	IHC	scores ≥ 4	32(2–129)	DFS	7
Ciocca	2009	America	166	Tissue	IHC	≥ 20% tumor cells staining	115(4–133)	RFS/OS	7
Yoon	2010	America	86	Tissue	IHC	weighted scores ≥ 1.8	NA	DFS	6
Gulbahce	2013	America	516	Tissue	IHC	≥ 1% tumor cells staining	81(3–140)	DFS	7
Hosoda	2014	Japan	289	Tissue	IHC	≥ 10% positive nuclear staining	66.2(3–114)	DFS	7
Hisamatsu	2015	Japan	214	Tissue	IHC	≥ 77.4% median value	58.1	DFS/OS	8
McCleskey	2015	America	72	Tissue	IHC	H-score > 210	55.2	RFS/OS	7
Min	2016	Korea	230	Tissue	IHC	immunoreactive score > 5	NA	RFS/OS	6

NOS score, Newcastle-Ottawa Scale score; NA, not available; H-score, Histo-score; DFS, disease-free survival; OS, overall survival; RFS, relapse-free survival; BCSS, breast cancer-specific survival; DRFS, distant relapse-free survival.

The baseline characteristics of each article are summarized in Table 1. All 10 studies were published between 2005 and 2016. Seven included Caucasian patient populations (five from America, one from Canada, and one from Portugal) and three included Asian patient populations (two from Japan and one from Korea). IHC was used to measure the protein expression level of GATA3 in breast cancer tissues in all studies, but each study has its own cut-off value to identify whether the expression level of GATA3 was high or not. Follow-up after surgery ranged from 1 to 222 months. TTP was used as an endpoint in all studies, while five also used OS as an endpoint. Moreover, several included studies mentioned a connection between expression of GATA3 with clinicopathological features; among them, four focused on the expression of ER, PR, Her-2 and nuclear grade, while three focused on tumor size and lymph node metastasis.

Effect of GATA3 protein expression level on survival and pathological features of breast cancer.

**Fig 2:**
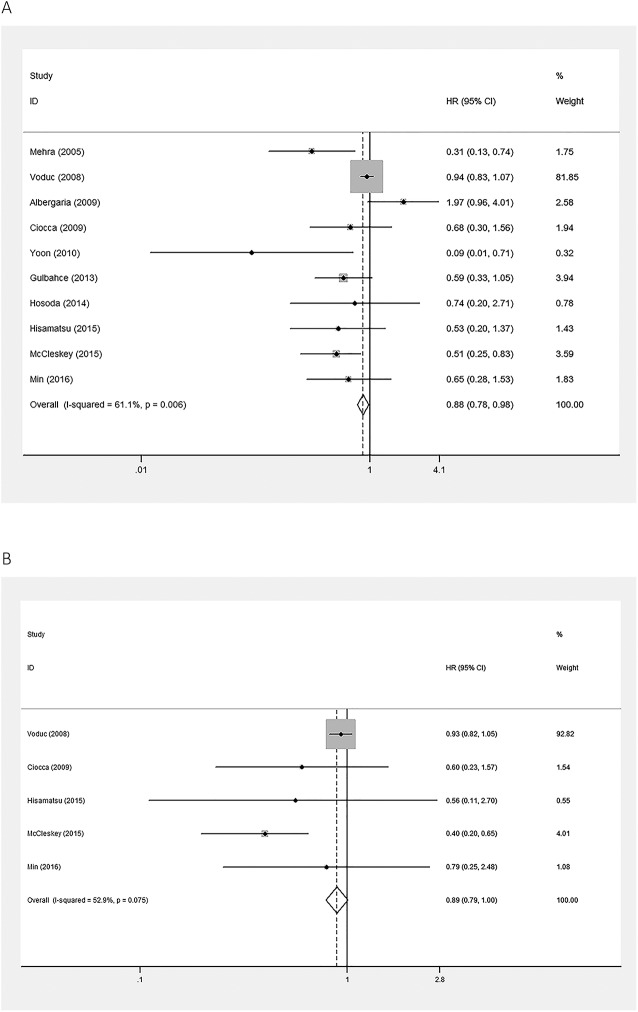
Forest plots of meta-analysis of the association between GATA3 expression and the prognoses of patients with breast cancer. A. Time to tumor progression (TTP); B. Overall survival (OS). Abbreviations: HR, hazard ratio; CI, confidence interval.

Pooled HRs of OS and TTP were used to illustrate survival of breast cancer patients in this meta-analysis. A random effects model was applied to calculate the pooled HR and 95% CI of TTP, which included 10 articles, as the heterogeneity test reported a P value of 0.006 and an I^2^ value of 61.1%. High expression of GATA3 was associated with better TTP of breast cancer (pooled HR = 0.671; 95% CI = 0.475–0.947; P = 0.023; Fig 2A). A fixed effects model was used to calculate the pooled HR and 95% CI of OS, which included five studies, as the heterogeneity test reported a P value of 0.075 and an I^2^ value of 52.9%. There was no association between expression of GATA3 and OS in breast cancer (pooled HR = 0.889; 95% CI = 0.789–1.001; P = 0.052; Fig 2B).

**Fig 3:**
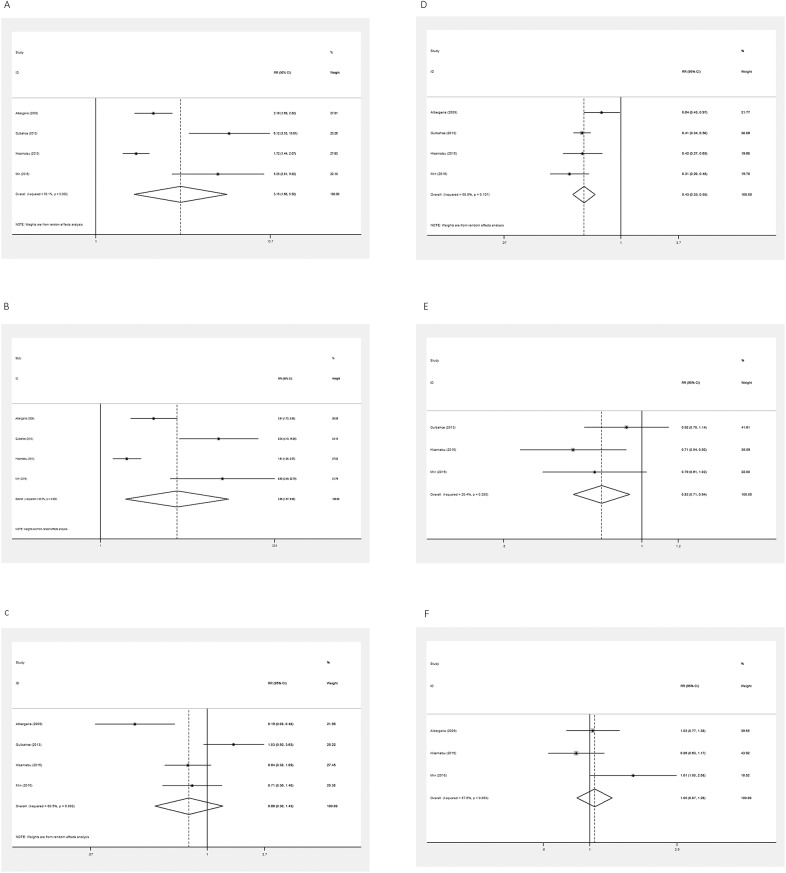
Forest plots of meta-analysis of the association between GATA3 expression and pathological features of patients with breast cancer. A. ER (+ vs.—); B. PR (+ vs.—); C. HER-2 (+ vs.—); D. nuclear grade (1 or 2 vs. 3); E. Tumor size (≤ 2 vs. > 2); F. Lymph node metastasis (negative vs. positive). Abbreviation: RR, risk ratio.

Pooled RRs were used to illustrate the association between GATA3 protein expression level and pathological features of breast cancer. Four studies were included in the meta-analysis of ER, PR, and HER-2 expression. Random effects models were used to calculate the pooled RR and 95% CI for existent heterogeneity (I^2^ = 93.1%, P = 0.000; I^2^ = 93.0%, P = 0.000; I^2^ = 80.5%, P = 0.002). High expression of GATA3 was associated with positive expression of ER and PR, but had no association with HER-2 (pooled RR = 3.155, 95% CI = 1.680–5.923, P = 0.000; pooled RR = 3.949, 95% CI = 1.567–9.954, P = 0.004; pooled RR = 0.659, 95% CI = 0.304–1.427, P = 0.290; Fig 3A, 3B and 3C).

Four studies were included in the meta-analysis of nuclear grade (1 or 2 vs. 3). A fixed effects model was used to calculate the pooled RR and 95% CI, as the heterogeneity test reported a P value of 0.107 and an I^2^ value of 50.8%. High expression of GATA3 was associated with lower nuclear grade (pooled RR = 0.435; 95% CI = 0.369–0.514; P = 0.000; Fig 3D).

Three studies were included in the meta-analysis of tumor size (≤2 vs. >2) and lymph node metastasis (negative vs. positive). A fixed effects model was used for tumor size and a random effects model was used for lymph node metastasis to calculate the pooled RR and 95% CI for the heterogeneity test report as follows: I^2^ = 20.4%, P = 0.285; I^2^ = 57.6%, P = 0.094. High expression of GATA3 was associated with smaller tumor size, but was unrelated to lymph node metastasis (pooled RR = 0.816, 95% CI = 0.709–0.940, P = 0.005; pooled RR = 0.907, 95% CI = 0.808–1.017, P = 0.611; Fig 3E and 3F)

### Publication bias and sensitivity analysis

**Fig 4:**
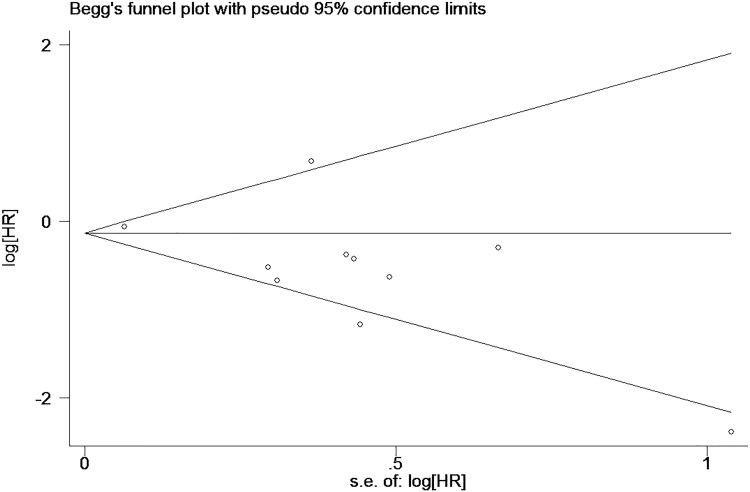
Begg’s funnel plots for the relationship between GATA3 expression and breast cancer.

**Fig 5:**
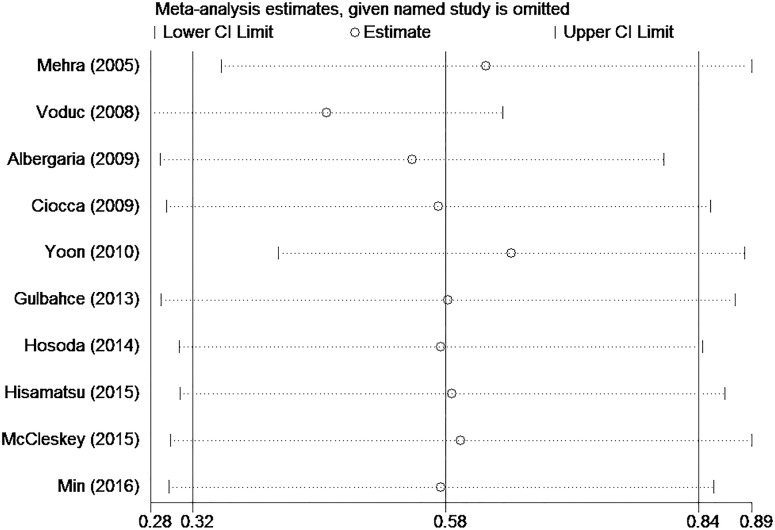
Sensitivity analysis to investigate the association between GATA3 expression and breast cancer prognosis.

We used Egger’s tests to estimate publication bias of the included studies for each meta-analysis that was statistically significant (TTP, expression of ER and PR, tumor size, and nuclear grade). Egger’s tests revealed non-significant values (all P > 0.05). The graphical output of the Begg’s funnel plots of the 10 included studies showed symmetry (Fig 4). In addition, sensitivity analysis was performed to assess the influence of individual studies on the meta-analyses. The significance of the pooled HRs was not affected by omitting any single study, which highlighted the lack of publication bias and supports the credibility of the results (Fig 5).

## Discussion

GATA3 is associated with a less aggressive phenotype and a better prognosis in patients with breast cancer [15–22, 24]. However, until now, no meta-analyses have been performed to evaluate the prognostic value of GATA3 protein expression in breast cancer patients. To our knowledge, this meta-analysis is the first comprehensive assessment of the literature focused on this topic. Our results indicate that high expression of GATA3 is associated with an unfavorable prognosis and is a predictive factor associated with TTP in breast cancer patients (pooled HR = 0.671; 95% CI = 0.475–0.947; P = 0.023) but is not significantly associated with OS (pooled HR = 0.889; 95% CI = 0.789–1.001; P = 0.052). To further explore the role of GATA3 in breast cancer patients, we performed subgroup analysis between GATA3 and clinicopathological parameters, finding that overexpression of GATA3 is associated with positive ER (pooled RR = 3.155; 95% CI = 1.680–5.923; P = 0.000), positive PR (pooled RR = 3.949; 95% CI = 1.567–9.954; P = 0.004), lower nuclear grade (pooled RR = 0.435; 95% CI = 0.369–0.514; P = 0.000), and smaller tumor size (pooled RR = 0.816; 95% CI = 0.709–0.940; P = 0.005), which is consistent with several studies that have been published [25–27].

To avoid bias from the method used to detect GATA3 protein expression, we excluded one study that used immunofluorescence staining [28]. Meanwhile, three studies that focused on GATA3 mRNA expression or GATA3 mutation by PCR or RT-PCR were excluded from this meta-analysis [29–31]. There is no doubt that comparing IHC-based protein expression with microarray-based gene-expression levels can lead to quite distinct conclusions. However, even at the gene level, the connection between GATA3 expression and survival of breast cancer patients exists. Thakkar et al. found that high expression of GATA3 mRNA improves prediction of RFS in estrogen receptor-positive and node-positive breast tumors [31] and Jiang et al. suggested that GATA3 mutations were correlated with improved OS in the entire population [29]; however, Liu et al. indicated that GATA3 mRNA expression, but not GATA3 mutation, is an independent predictor of prolonged progression-free survival (PFS) in ER-positive breast cancer patients who received first-line tamoxifen for recurrent disease [30]. The differences in results between these studies may be mainly due to differences in the detection method used, cut off value, and the subtype of breast cancer. Although there were differences in their findings, all found an association between GATA3 and survival at the gene level; however, this hypothesis requires further study.

Our meta-analysis suggests that GATA3 is a critical biomarker for predicting improved survival in breast cancer patients, which is the major finding of this study. Nevertheless, there are some limitations of this study that should be taken into consideration when interpreting patient outcomes. First, although we used a random-effects model when there was heterogeneity among the included studies, we could not avoid the effect of heterogeneity on the results. This heterogeneity may have been caused by varying patient characteristics or differences in the specific study designs in the different subtypes of breast cancer. Second, although we did not detect significant asymmetry via Begg’s funnel plots and found no evidence of publication bias via an Egger’s test, publication bias may have been inevitable as studies are more likely to be published if they have positive results than if they have negative results. Thus, the pooled results may be an overestimate. Third, the cut-off value of GATA3 protein expression varied between studies and the results are necessarily influenced by individual variations in interpretation by different observers or antibody, which is a major drawback and limits immediate clinical application. However, there is no standard cut-off value that is agreed upon, or used, by every one. Thus, to achieve a more convincing conclusion, further analysis using a larger sample size, a unified detection method, and adjusted individual data will be required.

In conclusion, this meta-analysis revealed that high expression of GATA3 in breast cancer is associated with improved TTP. Meanwhile, high GATA3 expression may be associated with positive ER, positive PR, smaller tumor size, and lower nuclear grade, all of which lead to improved survival.

## Supporting information

S1 File A full list of excluded articles and their reasons for exclusion. (DOC)

S1 Table PRISMA 2009 checklist. (DOC)
