# Supplementary material for: Prognostic and clinicopathological value of GATA binding protein 3 in breast cancer: A systematic review and meta-analysis
Source: PLoS One. 2017 Apr 10;12(4):e0174843. doi: 10.1371/journal.pone.0174843 (PMC5386271; doi:10.1371/journal.pone.0174843)
Supplement: S1 File — (DOC) [file pone.0174843.s001.doc]

**A full list of excluded articles and their reasons for exclusion**

1. duplicate publication[1-97];
2. non-English paper, non-human experiments [98];
3. conference abstract[45, 48, 62, 95, 99-243]；
4. letters , reviews, case reports, editorials, and comments [35, 65, 80, 97, 244-257];
5. not prognosis study about breast cancer[1-9, 11, 14-23, 25, 27, 28, 30-34, 37-39, 41-44, 46, 47, 50-61, 63, 64, 66, 68-70, 72, 74-76, 78, 79, 81, 83, 84, 89, 90, 93, 94, 258-389];
6. insufficient data regarding 95% confidence interval (95% CI) and risk ratios (RR), or the Kaplan-Meier curve could not be extracted[12, 13, 29, 36, 67, 73, 77, 82, 85, 91, 92, 390-399]

1. Hennequin C, Durdux C, Espie M, Balla-Mekias S, Housset M, Marty M, et al. High-dose-rate brachytherapy for early breast cancer: An ambulatory technique. International Journal of Radiation Oncology Biology Physics. 1999;45(1):85-90. doi: 10.1016/S0360-3016(99)00139-X. PubMed PMID: WOS:000081934500013.

2. Bertucci F, Houlgatte R, Benziane A, Granjeaud S, Adelaide J, Tagett R, et al. Gene expression profiling of primary breast carcinomas using arrays of candidate genes. Human molecular genetics. 2000;9(20):2981-91. doi: 10.1093/hmg/9.20.2981. PubMed PMID: WOS:000165965400007.

3. Baglan KL, Martinez AA, Frazier RC, Kini VR, Kestin LL, Chen PY, et al. The use of high-dose-rate brachytherapy alone after lumpectomy in patients with early-stage breast cancer treated with breast-conserving therapy. International Journal of Radiation Oncology Biology Physics. 2001;50(4):1003-11. doi: 10.1016/S0360-3016(01)01547-4. PubMed PMID: WOS:000169615100019.

4. Mayer A, Naszaly A, Patyanik M, Zarand P, Polgar I, Klinko T. Perioperative brachytherapy for pretreated chest wall recurrence of breast cancer. Strahlentherapie Und Onkologie. 2002;178(11):633-6. doi: 10.1007/s00066-002-0933-8. PubMed PMID: WOS:000179403500007.

5. Polgar C, Sulyok Z, Fodor J, Orosz Z, Major T, Takacsi-Nagy Z, et al. Sole brachytherapy of the tumor bed after conservative surgery for T1 breast cancer: Five-year results of a phase I-II study and initial findings of a randomized phase III trial. Journal of surgical oncology. 2002;80(3):121-8. doi: 10.1002/jso.10110. PubMed PMID: WOS:000176628200002.

6. Resch A, Potter R, Van Limbergen E, Biber E, Klein T, Fellner C, et al. Long-term results (10 years) of intensive breast conserving therapy including a high-dose and large-volume interstitial brachytherapy boost (LDR/HDR) for T1/T2 breast cancer. Radiotherapy and Oncology. 2002;63(1):47-58. doi: 10.1016/S0167-8140(02)00022-1. PubMed PMID: WOS:000177128000006.

7. Wazer DE, Berle L, Graham R, Chung M, Rothschild J, Graves T, et al. Preliminary results of a phase I/II study of HDR brachytherapy alone for T1/T2 breast cancer. International Journal of Radiation Oncology Biology Physics. 2002;53(4):889-97. doi: 10.1016/S0360-3016(02)02824-9. PubMed PMID: WOS:000176925400012.

8. Schaner ME, Ross DT, Ciaravino G, Sorlie T, Troyanskaya O, Diehn M, et al. Gene expression patterns in ovarian carcinomas. Molecular biology of the cell. 2003;14(11):4376-86. doi: 10.1091/mbc.E03-05-0279. PubMed PMID: WOS:000186738300005.

9. Ott OJ, Potter R, Hammer J, Hildebrandt G, Lotter M, Resch A, et al. Accelerated partial breast irradiation with iridium-192 multicatheter PDR/HDR brachytherapy: Preliminary results of the German-Austrian multicenter trial. Strahlentherapie und Onkologie. 2004;180(10):642-9.

10. Mehra R, Varambally S, Ding L, Shen R, Sabel MS, Ghosh D, et al. Identification of GATA3 as a breast cancer prognostic marker by global gene expression meta-analysis. Cancer research. 2005;65(24):11259-64. Epub 2005/12/17. doi: 10.1158/0008-5472.can-05-2495. PubMed PMID: 16357129.

11. Ott OJ, Potter R, Hildebrandt G, Hammer J, Lotter M, Beckmann MW, et al. Partial breast irradiation for early breast cancer: 3-year results of the German-Austrian phase II-trial. Rofo-Fortschritte Auf Dem Gebiet Der Rontgenstrahlen Und Der Bildgebenden Verfahren. 2005;177(7):962-7. doi: 10.1055/s-2005-858268. PubMed PMID: WOS:000230273900006.

12. Dolled-Filhart M, Ryden L, Cregger M, Jirstrom K, Harigopal M, Camp RL, et al. Classification of breast cancer using genetic algorithms and tissue microarrays. Clinical cancer research : an official journal of the American Association for Cancer Research. 2006;12(21):6459-68. Epub 2006/11/07. doi: 10.1158/1078-0432.ccr-06-1383. PubMed PMID: 17085660.

13. Oh DS, Troester MA, Usary J, Hu Z, He X, Fan C, et al. Estrogen-regulated genes predict survival in hormone receptor-positive breast cancers. Journal of clinical oncology : official journal of the American Society of Clinical Oncology. 2006;24(11):1656-64. Epub 2006/03/01. doi: 10.1200/jco.2005.03.2755. PubMed PMID: 16505416.

14. Roddiger SJ, Kolotas C, Filipowicz I, Kurek R, Kuner RP, Martin T, et al. Neoadjuvant interstitial high-dose-rate (HDR) brachytherapy combined with systemic chemotherapy in patients with breast cancer. Strahlentherapie Und Onkologie. 2006;182(1):22-9. doi: 10.1007/s0006-006-1454-7. PubMed PMID: WOS:000234943400004.

15. Wazer DE, Kaufman S, Cuttino L, DiPetrillo T, Arthur DW. Accelerated partial breast irradiation: An analysis of variables associated with late toxicity and long-term cosmetic outcome after high-dose-rate interstitial brachytherapy. International Journal of Radiation Oncology Biology Physics. 2006;64(2):489-95. doi: 10.1016/j.ijrobp.2005.06.028. PubMed PMID: WOS:000234883300021.

16. Wieners G, Pech M, Rudzinska M, Lehmkuhl L, Wlodarczyk W, Miersch A, et al. CT-guided interstitial brachytherapy in the local treatment of extrahepatic, extrapulmonary secondary malignancies. European Radiology. 2006;16(11):2586-93. doi: 10.1007/s00330-006-0241-2. PubMed PMID: WOS:000241608100023.

17. Budrukkar AN, Sarin R, Shrivastava SK, Deshpande DD, Dinshaw KA. Cosmesis, late sequelae and local control after breast-conserving therapy: Influence of type of tumour bed boost and adjuvant chemotherapy. Clinical oncology. 2007;19(8):596-603. doi: 10.1016/j.clon.2007.06.008. PubMed PMID: WOS:000250075000007.

18. Lovey K, Fodor J, Major T, Szabo E, Orosz Z, Sulyok Z, et al. Fat necrosis after partial-breast irradiation with brachytherapy or electron irradiation versus standard whole-breast radiotherapy - 4-year results of a randomized trial. International Journal of Radiation Oncology Biology Physics. 2007;69(3):724-31. doi: 10.1016/j.ijrobp.2007.03.055. PubMed PMID: WOS:000249943000011.

19. Manuylov NL, Smagulova FO, Tevosian SG. Fog2 excision in mice leads to premature mammary gland involution and reduced Esr1 gene expression. Oncogene. 2007;26(36):5204-13. doi: 10.1038/sj.onc.1210333. PubMed PMID: WOS:000248674200004.

20. Neumanova R, Petera J, Frgala T, Dusek L, Jarkovsky J, Kuricka R. Long-term outcome with interstitial brachytherapy boost in the treatment of women with early-stage breast cancer. Neoplasma. 2007;54(5):413-23. PubMed PMID: WOS:000251196500009.

21. Ott OJ, Hildebrandt G, Potter R, Hammer J, Lotter M, Resch A, et al. Accelerated partial breast irradiation with multi-catheter brachytherapy: Local control, side effects and cosmetic outcome for 274 patients. Results of the German-Austrian multi-centre trial. Radiotherapy and Oncology. 2007;82(3):281-6. doi: 10.1016/j.radonc.2006.08.028. PubMed PMID: WOS:000245413000006.

22. Polgar C, Fodor J, Major T, Nemeth G, Lovey K, Orosz Z, et al. Breast-conserving treatment with partial or whole breast irradiation for low-risk invasive breast carcinoma-5-year results of a randomized trial. International Journal of Radiation Oncology Biology Physics. 2007;69(3):694-702. doi: 10.1016/j.i.irobp.2007.04.022. PubMed PMID: WOS:000249943000007.

23. Arthur DW, Winter K, Kuske RR, Bolton J, Rabinovitch R, White J, et al. A Phase II trial of brachytherapy alone after lumpectomy for select breast cancer: Tumor control and survival outcomes of RTOG 95-17. International Journal of Radiation Oncology Biology Physics. 2008;72(2):467-73. doi: 10.1016/j.ijrobp.2007.12.056. PubMed PMID: WOS:000259329800023.

24. Albergaria A, Paredes J, Sousa B, Milanezi F, Carneiro V, Bastos J, et al. Expression of FOXA1 and GATA-3 in breast cancer: the prognostic significance in hormone receptor-negative tumours. Breast cancer research : BCR. 2009;11(3):R40. Epub 2009/06/25. doi: 10.1186/bcr2327. PubMed PMID: 19549328; PubMed Central PMCID: PMCPMC2716509.

25. Bollet MA, Savignoni A, De Koning L, Tran-Perennou C, Barbaroux C, Degeorges A, et al. Tumor aromatase expression as a prognostic factor for local control in young breast cancer patients after breast-conserving treatment. Breast Cancer Research. 2009;11(4). doi: 10.1186/bcr2343. PubMed PMID: WOS:000271414000017.

26. Ciocca V, Daskalakis C, Ciocca RM, Ruiz-Orrico A, Palazzo JP. The significance of GATA3 expression in breast cancer: a 10-year follow-up study. Human pathology. 2009;40(4):489-95. doi: 10.1016/j.humpath.2008.09.010. PubMed PMID: WOS:000264990200007.

27. Deblois G, Hall JA, Perry MC, Laganiere J, Ghahremani M, Park M, et al. Genome-wide identification of direct target genes implicates estrogen-related receptor alpha as a determinant of breast cancer heterogeneity. Cancer research. 2009;69(15):6149-57.

28. Dydensborg AB, Rose AAN, Wilson BJ, Grote D, Paquet M, Giguere V, et al. GATA3 inhibits breast cancer growth and pulmonary breast cancer metastasis. Oncogene. 2009;28(29):2634-42. doi: 10.1038/onc.2009.126. PubMed PMID: WOS:000268211200002.

29. Jacquemier J, Charafe-Jauffret E, Monville F, Esterni B, Extra JM, Houvenaeghel G, et al. Association of GATA3, P53, Ki67 status and vascular peritumoral invasion are strongly prognostic in luminal breast cancer. Breast cancer research : BCR. 2009;11(2):R23. Epub 2009/05/02. doi: 10.1186/bcr2249. PubMed PMID: 19405945; PubMed Central PMCID: PMCPMC2688952.

30. Nakshatri H, Badve S. FOXA1 in breast cancer. Expert Reviews in Molecular Medicine. 2009;11. doi: 10.1017/S1462399409001008. PubMed PMID: WOS:000276225400001.

31. Pei XH, Bai F, Smith MD, Usary J, Fan C, Pai SY, et al. CDK Inhibitor p18(INK4c) Is a Downstream Target of GATA3 and Restrains Mammary Luminal Progenitor Cell Proliferation and Tumorigenesis. Cancer cell. 2009;15(5):389-401. doi: 10.1016/j.ccr.2009.03.004. PubMed PMID: WOS:000265903200007.

32. Yoshida K, Nose T, Masuda N, Yamazaki H, Kotsuma T, Yoshida M, et al. Preliminary result of accelerated partial breast irradiation after breast-conserving surgery. Breast cancer (Tokyo, Japan). 2009;16(2):105-12. Epub 2008/09/23. doi: 10.1007/s12282-008-0067-7. PubMed PMID: 18807124.

33. Ademuyiwa FO, Thorat MA, Jain RK, Nakshatri H, Badve S. Expression of Forkhead- box protein A1, a marker of luminal A type breast cancer, parallels low Oncotype DX 21-gene recurrence scores. Modern Pathology. 2010;23(2):270-5. doi: 10.1038/modpathol.2009.172. PubMed PMID: WOS:000274233100013.

34. Bernardo GM, Lozada KL, Miedler JD, Harburg G, Hewitt SC, Mosley JD, et al. FOXA1 is an essential determinant of ER alpha expression and mammary ductal morphogenesis. Development. 2010;137(12):2045-54. doi: 10.1242/dev.043299. PubMed PMID: WOS:000278001500013.

35. Chou J, Provot S, Werb Z. GATA3 in Development and Cancer Differentiation: Cells GATA Have It! Journal of cellular physiology. 2010;222(1):42-9. doi: 10.1002/jcp.21943. PubMed PMID: WOS:000272528300007.

36. Demir H, Turna H, Can G, Ilvan S. Clinicopathologic and prognostic evaluation of invasive breast carcinoma molecular subtypes and GATA3 expression. Journal of Buon. 2010;15(4):774-82. PubMed PMID: WOS:000286539800024.

37. Polgar C, Janvary L, Major T, Somogyi A, Takacsi-Nagy Z, Frohlich G, et al. The role of high-dose-rate brachytherapy boost in breast-conserving therapy: Long-term results of the Hungarian National Institute of Oncology. Reports of Practical Oncology and Radiotherapy. 2010;15(1):1-7.

38. Rulli A, Barberini F, Scialpi M, Izzo L, D'Angeli I, Gori S, et al. Interstitial high-dose-rate brachytherapy after breast conserving surgery. Oncology reports. 2010;24(2):417-22. doi: 10.3892/or_00000874. PubMed PMID: WOS:000280050400016.

39. Yan W, Cao QJ, Arenas RB, Bentley B, Shao R. GATA3 Inhibits Breast Cancer Metastasis through the Reversal of Epithelial-Mesenchymal Transition. Journal of Biological Chemistry. 2010;285(18):14042-51. doi: 10.1074/jbc.M110.105262. PubMed PMID: WOS:000276987700077.

40. Yoon NK, Maresh EL, Shen D, Elshimali Y, Apple S, Horvath S, et al. Higher levels of GATA3 predict better survival in women with breast cancer. Human pathology. 2010;41(12):1794-801. Epub 2010/11/17. doi: 10.1016/j.humpath.2010.06.010. PubMed PMID: 21078439; PubMed Central PMCID: PMCPMC2983489.

41. Chichel A, Skowronek J, Kanikowski M. Thermal boost combined with interstitial brachytherapy in breast conserving therapy - Assessment of early toxicity. Reports of Practical Oncology and Radiotherapy. 2011;16(3):87-94.

42. Dooley WC, Wurzer JC, Megahy M, Schreiber G, Roy T, Proulx G, et al. Electronic brachytherapy as adjuvant therapy for early stage breast cancer: a retrospective analysis. OncoTargets and therapy. 2011;4:13-20. doi: 10.2147/OTT.S15297. PubMed PMID: WOS:000286673800001.

43. Kung HN, Marks JR, Chi JT. Glutamine Synthetase Is a Genetic Determinant of Cell Type-Specific Glutamine Independence in Breast Epithelia. Plos Genetics. 2011;7(8). doi: 10.1371/journal.pgen.1002229. PubMed PMID: WOS:000294297000025.

44. McHaffie DR, Patel RR, Adkison JB, Das RK, Geye HM, Cannon GM. Outcomes after Accelerated Partial Breast Irradiation in Patients with Astro Consensus Statement Cautionary Features. International Journal of Radiation Oncology Biology Physics. 2011;81(1):46-51. doi: 10.1016/j.ijrobp.2010.05.011. PubMed PMID: WOS:000294093300007.

45. Ott OJ, Hildebrandt G, Potter R, Hammer J, Hindemith M, Resch A, et al. Accelerated Partial Breast Irradiation with Interstitial Implants: Risk Factors Associated with Increased Local Recurrence. International Journal of Radiation Oncology Biology Physics. 2011;80(5):1458-63. doi: 10.1016/j.ijrobp.2010.04.032. PubMed PMID: WOS:000293207600026.

46. Pignol JP, Keller BM, Ravi A. Doses to internal organs for various breast radiation techniques - implications on the risk of secondary cancers and cardiomyopathy. Radiation Oncology. 2011;6. doi: 10.1186/1748-717X-6-5. PubMed PMID: WOS:000286585300001.

47. Shao R, Cao QJ, Arenas RB, Bigelow C, Bentley B, Yan W. Breast cancer expression of YKL-40 correlates with tumour grade, poor differentiation, and other cancer markers. British journal of cancer. 2011;105(8):1203-9. doi: 10.1038/bjc.2011.347. PubMed PMID: WOS:000296144900016.

48. Shen D, Yoon NK, Maresh EL, Elshimali Y, Apple S, Horvath S, et al. Higher Levels of GATA3 Predict Better Survival in Women with Breast Cancer. Laboratory Investigation. 2011;91:63A-A. PubMed PMID: WOS:000287011400255.

49. Banerji S, Cibulskis K, Rangel-Escareno C, Brown KK, Carter SL, Frederick AM, et al. Sequence analysis of mutations and translocations across breast cancer subtypes. Nature. 2012;486(7403):405-9.

50. Chu IM, Michalowski AM, Hoenerhoff M, Szauter KM, Luger D, Sato M, et al. GATA3 inhibits lysyl oxidase-mediated metastases of human basal triple-negative breast cancer cells. Oncogene. 2012;31(16):2017-27. doi: 10.1038/onc.2011.382. PubMed PMID: WOS:000303008600003.

51. Dolezel M, Stastny K, Odrazka K, Vanasek J, Kohlova T, Dvorakova D, et al. Perioperative interstitial CT-based brachytherapy boost in breast cancer patients with breast conservation after neoadjuvant chemotherapy. Neoplasma. 2012;59(5):494-9. doi: 10.4149/neo_2012_063. PubMed PMID: WOS:000307860500003.

52. Guinot JL, Tortajada MI, Carrascosa M, Crispin V, Otero A, Rios B, et al. Ten-year results of a phase II study with a single fraction of high-dose-rate brachytherapy (FAST-boost) after whole breast irradiation in invasive breast carcinoma. Clinical & Translational Oncology. 2012;14(2):109-15. doi: 10.1007/s12094-012-0769-3. PubMed PMID: WOS:000301122200005.

53. Tkocz D, Crawford NT, Buckley NE, Berry FB, Kennedy RD, Gorski JJ, et al. BRCA1 and GATA3 corepress FOXC1 to inhibit the pathogenesis of basal-like breast cancers. Oncogene. 2012;31(32):3667-78. doi: 10.1038/onc.2011.531. PubMed PMID: WOS:000307653800002.

54. Chou J, Lin JH, Brenot A, Kim JW, Provot S, Werb Z. GATA3 suppresses metastasis and modulates the tumour microenvironment by regulating microRNA-29b expression. Nature Cell Biology. 2013;15(2):201-13. doi: 10.1038/ncb2672. PubMed PMID: WOS:000314856700011.

55. Ciriello G, Sinha R, Hoadley KA, Jacobsen AS, Reva B, Perou CM, et al. The molecular diversity of Luminal A breast tumors. Breast cancer research and treatment. 2013;141(3):409-20. doi: 10.1007/s10549-013-2699-3. PubMed PMID: WOS:000326041100009.

56. Jardim DLF, Conley A, Subbiah V. Comprehensive characterization of malignant phyllodes tumor by whole genomic and proteomic analysis: biological implications for targeted therapy opportunities. Orphanet Journal of Rare Diseases. 2013;8. doi: 10.1186/1750-1172-8-112. PubMed PMID: WOS:000323391600001.

57. Johansson I, Ringner M, Hedenfalk I. The Landscape of Candidate Driver Genes Differs between Male and Female Breast Cancer. PloS one. 2013;8(10). doi: 10.1371/journal.pone.0078299. PubMed PMID: WOS:000326037000095.

58. Polgar C, Fodor J, Major T, Sulyok Z, Kasler M. Breast-conserving therapy with partial or whole breast irradiation: Ten-year results of the Budapest randomized trial. Radiotherapy and Oncology. 2013;108(2):197-202. doi: 10.1016/j.radonc.2013.05.008. PubMed PMID: WOS:000326139300003.

59. Powers GL, Rajbhandari P, Solodin NM, Bickford B, Alarid ET. The proteasome inhibitor bortezomib induces an inhibitory chromatin environment at a distal enhancer of the estrogen receptor-alpha gene. PloS one. 2013;8(12):no pagination.

60. Sedlmayer F, Zehentmayr F, Fastner G. Partial breast re-irradiation for local recurrence of breast carcinoma: Benefit and long term side effects. Breast (Edinburgh, Scotland). 2013;22:S141-S6. doi: 10.1016/j.breast.2013.07.026. PubMed PMID: WOS:000326312100028.

61. So JS, Epstein JI. GATA3 expression in paragangliomas: a pitfall potentially leading to misdiagnosis of urothelial carcinoma. Modern Pathology. 2013;26(10):1365-70. doi: 10.1038/modpathol.2013.76. PubMed PMID: WOS:000325214000010.

62. Surowiecka M, Blair C, Knapp D, Varghese L, Gulbahce E. Significance of GATA3 Expression in Outcomes of Breast Cancer Patients Who Received Systemic Chemo and/or Hormonal Therapy and Clinicopathological Features of GATA3 Positive Tumors. Modern Pathology. 2013;26:71A-A. PubMed PMID: WOS:000314444400288.

63. Theodorou V, Stark R, Menon S, Carroll JS. GATA3 acts upstream of FOXA1 in mediating ESR1 binding by shaping enhancer accessibility. Genome Research. 2013;23(1):12-22. doi: 10.1101/gr.139469.112. PubMed PMID: WOS:000312963400002.

64. Tselis N, Chatzikonstantinou G, Kolotas C, Milickovic N, Baltas D, Zamboglou N. Computed tomography-guided interstitial high dose rate brachytherapy for centrally located liver tumours: a single institution study. European Radiology. 2013;23(8):2264-70. doi: 10.1007/s00330-013-2816-z. PubMed PMID: WOS:000321523500027.

65. Vasconcelos ALC, Nunes B, Duarte C, Mendonca V, Ribeiro J, Jorge M, et al. Tamoxifen in breast cancer ipse dixit in uterine malignant mixed Mullerian tumor and sarcoma-A report of 8 cases and review of the literature. Reports of Practical Oncology and Radiotherapy. 2013;18(5):251-60.

66. Yamazaki H, Yoshida K, Yoshioka Y, Shimizutani K, Furukawa S, Koizumi M, et al. High dose rate brachytherapy for oral cancer. Journal of Radiation Research. 2013;54(1):1-17. doi: 10.1093/jrr/rrs103. PubMed PMID: WOS:000313127200001.

67. Yu KD, Zhu R, Zhan M, Rodriguez AA, Yang W, Wong S, et al. Identification of prognosis-relevant subgroups in patients with chemoresistant triple-negative breast cancer. Clinical cancer research : an official journal of the American Association for Cancer Research. 2013;19(10):2723-33. Epub 2013/04/04. doi: 10.1158/1078-0432.ccr-12-2986. PubMed PMID: 23549873; PubMed Central PMCID: PMCPMC3655097.

68. Castilla MA, Lopez-Garcia MA, Atienza MR, Rosa-Rosa JM, Diaz-Martin J, Pecero ML, et al. VGLL1 expression is associated with a triple-negative basal-like phenotype in breast cancer. Endocrine-related cancer. 2014;21(4):587-99. doi: 10.1530/ERC-13-0485. PubMed PMID: WOS:000344788200026.

69. Cohen H, Ben-Hamo R, Gidoni M, Yitzhaki I, Kozol R, Zilberberg A, et al. Shift in GATA3 functions, and GATA3 mutations, control progression and clinical presentation in breast cancer. Breast Cancer Research. 2014;16(6). doi: 10.1186/s13058-014-0464-0. PubMed PMID: WOS:000349885800004.

70. Frietze S, O'Geen H, Littlepage LE, Simion C, Sweeney CA, Farnham PJ, et al. Global analysis of ZNF217 chromatin occupancy in the breast cancer cell genome reveals an association with ERalpha. Bmc Genomics. 2014;15. doi: 10.1186/1471-2164-15-520. PubMed PMID: WOS:000338250400001.

71. Hosoda M, Yamamoto M, Nakano K, Hatanaka KC, Takakuwa E, Hatanaka Y, et al. Differential expression of progesterone receptor, FOXA1, GATA3, and p53 between pre- and postmenopausal women with estrogen receptor-positive breast cancer. Breast cancer research and treatment. 2014;144(2):249-61. doi: 10.1007/s10549-014-2867-0. PubMed PMID: WOS:000332959000005.

72. Izzo F, Mercogliano F, Venturutti L, Tkach M, Inurrigarro G, Schillaci R, et al. Progesterone receptor activation downregulates GATA3 by transcriptional repression and increased protein turnover promoting breast tumor growth. Breast Cancer Research. 2014;16(6). doi: 10.1186/s13058-014-0491-x. PubMed PMID: WOS:000349885800029.

73. Jiang YZ, Yu KD, Zuo WJ, Peng WT, Shao ZM. GATA3 mutations define a unique subtype of luminal-like breast cancer with improved survival. Cancer. 2014;120(9):1329-37. doi: 10.1002/cncr.28566. PubMed PMID: WOS:000334657100008.

74. Kabir NN, Ronnstrand L, Kazi JU. Keratin 19 expression correlates with poor prognosis in breast cancer. Molecular biology reports. 2014;41(12):7729-35. doi: 10.1007/s11033-014-3684-6. PubMed PMID: WOS:000349005800002.

75. Kim Y, Trombetta MG. Dosimetric evaluation of multilumen intracavitary balloon applicator rotation in high-dose-rate brachytherapy for breast cancer. J Appl Clin Med Phys. 2014;15(1):4429. Epub 2014/01/16. doi: 10.1120/jacmp.v15i1.4429. PubMed PMID: 24423837.

76. Lee J, Tiwari A, Shum V, Mills GB, Mancini MA, Igoshin OA, et al. Unraveling the regulatory connections between two controllers of breast cancer cell fate. Nucleic acids research. 2014;42(11):6839-49. doi: 10.1093/nar/gku360. PubMed PMID: WOS:000338769400012.

77. Miettinen M, McCue PA, Sarlomo-Rikala M, Rys J, Czapiewski P, Wazny K, et al. GATA3: a multispecific but potentially useful marker in surgical pathology: a systematic analysis of 2500 epithelial and nonepithelial tumors. The American journal of surgical pathology. 2014;38(1):13-22. Epub 2013/10/23. doi: 10.1097/PAS.0b013e3182a0218f. PubMed PMID: 24145643; PubMed Central PMCID: PMCPMC3991431.

78. Tudoran O, Virtic O, Balacescu L, Pop L, Dragla F, Eniu A, et al. Differential Peripheral Blood Gene Expression Profile Based on Her2 Expression on Primary Tumors of Breast Cancer Patients. PloS one. 2014;9(7). doi: 10.1371/journal.pone.0102764. PubMed PMID: WOS:000339993700018.

79. Abduljabbar R, Negm OH, Lai CF, Jerjees DA, Al-Kaabi M, Hamed MR, et al. Clinical and biological significance of glucocorticoid receptor (GR) expression in breast cancer. Breast cancer research and treatment. 2015;150(2):335-46. doi: 10.1007/s10549-015-3335-1. PubMed PMID: WOS:000352202200010.

80. Balgobind BV, Koedooder K, Zuniga DO, Fajardo RD, Rasch CRN, Pieters BR. A review of the clinical experience in pulsed dose rate brachytherapy. British Journal of Radiology. 2015;88(1055). doi: 10.1259/bjr.20150310. PubMed PMID: WOS:000366681700015.

81. Budrukkar A, Gurram L, Upreti RR, Munshi A, Jalali R, Badwe R, et al. Clinical outcomes of prospectively treated 140 women with early stage breast cancer using accelerated partial breast irradiation with 3 dimensional computerized tomography based brachytherapy. Radiotherapy and Oncology. 2015;115(3):349-54. doi: 10.1016/j.radonc.2015.03.002. PubMed PMID: WOS:000358804600010.

82. Espinosa I, Gallardo A, D'Angelo E, Mozos A, Lerma E, Prat J. Simultaneous Carcinomas of the Breast and Ovary: Utility of Pax-8, WT-1, and GATA3 for Distinguishing Independent Primary Tumors from Metastases. International Journal of Gynecological Pathology. 2015;34(3):257-65. PubMed PMID: WOS:000352640800008.

83. Glubb DM, Maranian MJ, Michailidou K, Pooley KA, Meyer KB, Kar S, et al. Fine-Scale Mapping of the 5q11.2 Breast Cancer Locus Reveals at Least Three Independent Risk Variants Regulating MAP3K1. American Journal of Human Genetics. 2015;96(1):5-20. doi: 10.1016/j.ajhg.2014.11.009. PubMed PMID: WOS:000347707800001.

84. Guinot JL, Baixauli-Perez C, Soler P, Tortajada MI, Moreno A, Santos MA, et al. High-Dose-Rate Brachytherapy Boost Effect on Local Tumor Control in Young Women With Breast Cancer. International Journal of Radiation Oncology Biology Physics. 2015;91(1):165-71. doi: 10.1016/j.ijrobp.2014.09.024. PubMed PMID: WOS:000346415500024.

85. Hattori Y, Yoshida A, Yoshida M, Takahashi M, Tsuta K. Evaluation of androgen receptor and GATA binding protein 3 as immunohistochemical markers in the diagnosis of metastatic breast carcinoma to the lung. Pathology international. 2015;65(6):286-92. doi: 10.1111/pin.12278. PubMed PMID: WOS:000355651400002.

86. Hisamatsu Y, Tokunaga E, Yamashita N, Akiyoshi S, Okada S, Nakashima Y, et al. Impact of GATA-3 and FOXA1 expression in patients with hormone receptor-positive/HER2-negative breast cancer. Breast cancer (Tokyo, Japan). 2015;22(5):520-8. Epub 2014/01/15. doi: 10.1007/s12282-013-0515-x. PubMed PMID: 24415069.

87. Li R, Campos J, Iida J. A gene regulatory program in human breast cancer. Genetics. 2015;201(4):1341-8.

88. McCleskey BC, Penedo TL, Zhang K, Hameed O, Siegal GP, Wei S. GATA3 Expression in Advanced Breast Cancer: Prognostic Value and Organ-Specific Relapse. American journal of clinical pathology. 2015;144(5):756-63. doi: 10.1309/AJCP5MMR1FJVVIPK. PubMed PMID: WOS:000363226300010.

89. Mohammadinejad P, Arya P, Esfandbod M, Kaviani A, Najafi M, Kashani L, et al. Celecoxib Versus Diclofenac in Mild to Moderate Depression Management Among Breast Cancer Patients: A Double-Blind, Placebo-Controlled, Randomized Trial. Annals of Pharmacotherapy. 2015;49(9):953-61. doi: 10.1177/1060028015592215. PubMed PMID: WOS:000360031600001.

90. Perkins SM, Bales C, Vladislav T, Althouse S, Miller KD, Sandusky G, et al. TFAP2C expression in breast cancer: correlation with overall survival beyond 10 years of initial diagnosis. Breast cancer research and treatment. 2015;152(3):519-31. doi: 10.1007/s10549-015-3492-2. PubMed PMID: WOS:000358661600006.

91. Thakkar A, Raj H, Ravishankar, Muthuvelan B, Balakrishnan A, Padigaru M. High Expression of Three-Gene Signature Improves Prediction of Relapse-Free Survival in Estrogen Receptor-Positive and Node-Positive Breast Tumors. Biomarker insights. 2015;10:103-12. doi: 10.4137/BMIMI.S30559. PubMed PMID: WOS:000368578900001.

92. Wendroth SM, Mentrikoski MJ, Wick MR. GATA3 expression in morphologic subtypes of breast carcinoma: a comparison with gross cystic disease fluid protein 15 and mammaglobin. Annals of diagnostic pathology. 2015;19(1):6-9. doi: 10.1016/j.anndiagpath.2014.12.001. PubMed PMID: WOS:000349506100002.

93. Werner S, Brors B, Eick J, Marques E, Pogenberg V, Parret A, et al. Suppression of early hematogenous dissemination of human breast cancer cells to bone marrow by retinoic Acid-induced 2. Cancer discovery. 2015;5(5):506-19. Epub 2015/02/27. doi: 10.1158/2159-8290.cd-14-1042. PubMed PMID: 25716347.

94. Bitter SM, Heffron-Cartwright P, Wennerstrom C, Weatherford J, Einstein D, Keiler LC. WBRT vs. APBI: an interim report of patient satisfaction and outcomes. Journal of Contemporary Brachytherapy. 2016;8(1):17-22. doi: 10.5114/jcb.2016.57816. PubMed PMID: WOS:000372138300003.

95. Kim DH, Min KW, Do SI, Chae SW, Sohn JH. Negative association between GATA3 and fascin could predict relapse-free and overall survival in patients with breast cancer. Laboratory Investigation. 2016;96:51A.

96. Min KW, Kim DH, Do SI, Chae SW, Kim K, Sohn JH, et al. Negative association between GATA3 and fascin could predict relapse-free and overall survival in patients with breast cancer. Virchows Archiv. 2016;468(4):409-16. doi: 10.1007/s00428-015-1894-5. PubMed PMID: WOS:000374312900004.

97. Tempfer CB, El Fizazi N, Ergonenc H, Solass W. Metastasis of ovarian cancer to the breast: A report of two cases and a review of the literature. Oncology letters. 2016;11(6):4008-12. doi: 10.3892/ol.2016.4514. PubMed PMID: WOS:000377626700075.

98. Bao Y, Zhong ZX, Cui G, Guo L, Wang ZF. [Roles of trichorhinophalangeal syndrome-1 gene in normal breast development and breast cancer]. Zhongguo yi xue ke xue yuan xue bao Acta Academiae Medicinae Sinicae. 2013;35(1):121-4. Epub 2013/03/09. doi: 10.3881/j.issn.1000-503X.2013.01.023. PubMed PMID: 23469802.

99. Mehra R, Shen R, Varambally S, Chinnaiyan AM, Kleer CG. GATA3 expression in breast cancer: A strong independent predictor of survival. Modern Pathology. 2005;18:43A-A. PubMed PMID: WOS:000226117900185.

100. Alam N, Taylor C, Mandall P, James L, Logue J, Wylie J. Acute toxicity after single fraction high dose rate brachytherapy with external beam radiotherapy in intermediate and high risk prostate cancer. Clinical oncology. 2009;21(10):799.

101. Jauffret EA, Boher J, Esterni B, Rousseau F, Jacquemier J, Viens P. Breast cancer in the elderly: Histoclinial features and phenotypical characterization on tissue microarrays (TMA). Cancer research. 2009;69(2 Suppl. S):no pagination.

102. Picarsic J, Brufsky A, Onisko A, Chivukula M. Predictors of invasive breast cancer or DCIS recurrence in estrogen receptor positive (ER+) and estrogen receptor negative (ER-) ductal carcinoma in situ (DCIS) patients with and without associated invasive carcinoma (IC). Journal of Clinical Oncology. 2009;27(15 SUPPL. 1):e11523.

103. Swampillai A, Lewis R, Payne H. Long term follow-up of patients receiving high dose rate (HDR) brachytherapy boost in combination with external beam radiotherapy treatment (EBRT) for high risk prostate cancer: Relapse rate and toxicity. Clinical oncology. 2009;21(10):801.

104. Anonymous. Abstracts of the 31st Annual Meeting of the American Brachytherapy Society. Brachytherapy. 2010;9:no pagination.

105. Casquero Ocio F, Diaz Gavela AA, Del Hoyo O, Muruzabal Zaldivar I, Cacicedo Fernandez De Bobadilla J, Gomez-Iturriaga A, et al. Brachytherapy boost with high dose rate in conservative treatment of breast cancer. Radiotherapy and Oncology. 2010;96:S231.

106. Chen PY, Martinez AA, Wallace M, Mitchell C, Ghilezan MI, Brabbins D, et al. Comparative analysis of two distinct time-dose-fractionation schemes [2-day vs 5-day] for delivery of accelerated partial breast irradiation via the MammoSite RTS applicator. Brachytherapy. 2010;9:S31.

107. Di Palma A, Maranzano E, Casale M, Chirico L, Italian M, Anselmo P, et al. High-dose rate brachytherapy (HDR-BRT) for early breast cancer: Clinical and dosimetric results of a Phase II trial. Radiotherapy and Oncology. 2010;96:S86-S7.

108. Dolinska Z, Lukacko P, Chovanova V, Pobijakova M, Dolinsky J, Salek T. Intraluminal HDR 192lR brachytherapy as palliative treatment in patients with endobronchial metastases from breast cancer - NCI Slovakia experience. Radiotherapy and Oncology. 2010;96:S217.

109. Garcia Zamora I, Gutierrez Miguelez C, Pera Fabregas J, Navarro Martin A, Martinez Perez E, Navarro Perez V, et al. Partial breast irradiation with brachytherapy for breast cancer: The experience of the Catalan Institute of Oncology. Radiotherapy and Oncology. 2010;96:S242.

110. Guix B, Lejarcegui JA, Tello JI, Zanon G, Del-Campo JM, Milla A, et al. Partial breast brachytherapy after lumpectomy as salvage treatment for local recurrences after conservative treatment of breast cancer: Eighteen-year results of a non-randomized comparison with mastectomy. Brachytherapy. 2010;9:S31.

111. Gutierrez Miguelez C, Pera J, Garcia Zamora I, Navarro V, Navarro-Martin A, Martinez Perez E. Partial breast irradiation with brachytherapy for breast cancer: The experience of Institut Catala D'oncologia. Radiotherapy and Oncology. 2010;96:S221-S2.

112. Haro S, Mark R, Anderson P, Akins R, Nair M. Interstitial high dose rate (HDR) brachytherapy for breast cancer in women < 50 years of age compared to :> 50 years of age. A report of 264 cases using multi-catheter technique. Radiotherapy and Oncology. 2010;96:S238-S9.

113. Mark RJ, Anderson PJ, Akins RS, Nair M. Interstitial high-dose-rate brachytherapy for breast cancer in women <50 years of age compared to >50 years of age: Median 6-year followup in 251 cases using multi-catheter technique. Brachytherapy. 2010;9:S82.

114. Muthukaruppan A, Michael BA, Miller LD, Tamada Y, Allen TM, Woad KJ, et al. The role of oestrogen signalling in breast cancer. Annals of Oncology. 2010;21:iv54-iv5.

115. Ott O, Hildebrandt G, Potter R, Hammer J, Hindemith M, Resch A, et al. Accelerated partial breast irradiation with interstitial implants: Risk factors associated with increased local recurrence. Strahlentherapie und Onkologie. 2010;186:37.

116. Picarsic J, Brufsky A, Ahrendt G, Onisko A, Chivukula M. Role of transcription factors [FOXA1, GATA-3] in predicting outcome in (ER+) and (ER-) ductal carcinoma-in-situ (DCIS) patients with and without invasive carcinoma (IC): A retrospective subset analysis. Laboratory Investigation. 2010;90:66A.

117. Pignol JP, Keller BM, Ravi A. Scader dose to body organs for various breast irradiation techniques. Radiotherapy and Oncology. 2010;96:S5-S6.

118. Rodriguez Perez A, Saez Garrido JDD, Samper Ots PM, Lopez Carrizosa MC, Zapatero Ortuno J, Lopez Gonzalez M, et al. Early stage breast cancer conserving treatment: High dose rate brachytherapy boost to the tumor bed. Radiotherapy and Oncology. 2010;96:S235.

119. Strnad V, Hildebrandt G, Potter R, Hammer J, Lotter M, Fietkau R, et al. Accelerated partial breast irradiation after breast conserving surgery for early breast cancer: 5- and 8-year results of German-Austrian phase II trial. Radiotherapy and Oncology. 2010;96:S18-S9.

120. Tokunaga E, Hisamatsu Y, Okada S, Yamashita N, Nakashima Y, Saeki H, et al. Expression of forkhead-box protein A1 (FOXA1) as a significant prognostic and predictive marker for ER-positive breast cancer. Journal of Clinical Oncology. 2010;28(15 SUPPL. 1):no pagination.

121. Zhong J, Liao J, Yao L, Liu J, Stark J, Lees-Miller S, et al. A PP6-phosphatase complex is required for homology-directed repair of DNA double-strand breaks. Cancer research. 2010;70(8 SUPPL. 1):no pagination.

122. Andres SA, Wittliff JL. Relationship between gene and tumor marker expression in primary breast carcinoma and lymph node metastases of the same patient. Cancer research. 2011;71(8 SUPPL. 1):no pagination.

123. Budrukkar A, Sarin R, Jalali R, Munshi A, Badwe R, Seth T, et al. Five year clinical outcome in 109 women with clinically palpable tumours (1-3 cm) treated with accelerated partial breast irradiation using interstitial brachytherapy. European Journal of Cancer. 2011;47:S367.

124. Garsa AA, DeWees T, Zoberi I. Fat necrosis after breast conserving surgery and high dose rate interstitial brachytherapy for earlystage breast cancer. International Journal of Radiation Oncology Biology Physics. 2011;81(2 SUPPL. 1):S234-S5.

125. Gipson A, Mark R, Akins R, Nair M. Interstitial high dose rate (HDR) brachytherapy for breast cancer in women < 50 years of age compared to > 50 years of age : Median 6 year follow-up in 280 cases using multi-catheter technique. Radiotherapy and Oncology. 2011;99:S253.

126. Guinot JL, Carrascosa M, Tortajada-Azcutia M, Santos M, Soler P, Contreras VC, et al. Phase ii study with a single fraction of HDR brachytherapy (fast-boost) after whole breast irradiation in early breast cancer. Long-term results. Radiotherapy and Oncology. 2011;99:S254.

127. Guix B, Lejarcegui J, Tello J, Zanon G, Del-Campo J, Rubio D, et al. Partial breast brachytherapy after lumpectomy as salvage treatment of local recurrences after conservative treatment of breast cancer: Eighteen-year results of a non-randomized comparison with mastectomy. International Journal of Radiation Oncology Biology Physics. 2011;81(2 SUPPL. 1):S219.

128. Guix B, Lejarcegui JA, Tello JI, Guix I, Zanon G, Quinzanos L, et al. Accelerated partial breast brachytherapy after lumpectomy as salvage treatment for local recurrences after conservative treatment of breast cancer: Eighteen-year results of a nonrandomized comparison with mastectomy. Journal of Clinical Oncology. 2011;29(27 SUPPL. 1):no pagination.

129. Hisamatsu Y, Tokunaga E, Akiyoshi S, Okada S, Yamashita N, Oki E, et al. The expression of GATA-3 and FOXA1 in breast cancer: The biomarkers of hormone sensitivity in luminal-type tumors. Journal of Clinical Oncology. 2011;29(15 SUPPL. 1):no pagination.

130. Loessl K, Clhorlc N, Isaak B, Terrlblllnl D, Aebersold DM. Accelerated partial breast irradiation: A dosimetric comparison of peri-and postoperative implants. Radiotherapy and Oncology. 2011;99:S250.

131. Lukacko P, Dolinska Z, Chovanova V. HDR endobronchial brachytherapy for patients with metastatic breast cancer. Journal of Contemporary Brachytherapy. 2011;3(3):170-1.

132. Mark RJ, White D, Akins R, Mutyala S, Nair M. Interstitial high dose rate (HDR) brachytherapy for breast cancer in women<50 years of age compared to . 50 Years of age: Median 6 year follow-up in 283 cases using multi-catheter technique. International Journal of Radiation Oncology Biology Physics. 2011;81(2 SUPPL. 1):S241.

133. McHaffie DR, Patel RR, Das RK, Geye HM, Cannon GM. Pathologic features associated with locoregional recurrence following brachytherapy-based accelerated partial breast irradiation. Brachytherapy. 2011;10:S39-S40.

134. Palumbo I, Farneti A, Raymondi C, Margaritelli M, Lancellotta V, Falcinelli L, et al. Interstitial hdr brachytherapy for PBI in early breast cancer: Results after a minimum follow-up of three years. Radiotherapy and Oncology. 2011;99:S301.

135. Peddada AV, Pomeranke LK, Young J, Bulz G, Monroe AT. Outcomes following a community based program of accelerated partial breast irradiation using HDR brachytherapy in early stage breast cancer. Brachytherapy. 2011;10:S42.

136. Petrovsky A, Gevorkyan V, Nechushkin M. HDR-brachytherapy for internal mammary lymph nodes in breast cancer patients. Journal of Contemporary Brachytherapy. 2011;3(1):51.

137. Petrovsky A, Gevorkyan V, Zaytseva A, Nechushkin M. Different types of brachytherapy at the internal mammary nodes in breast cancer patients. Brachytherapy. 2011;10:S43.

138. Pignol J, Keller BM, Rakovitch E, Ravi A, Chan G, Mashouf S. Long-term results of adjuvant accelerated partial breast irradiation using permanent 103-palladium seed implants. International Journal of Radiation Oncology Biology Physics. 2011;81(2 SUPPL. 1):S226-S7.

139. Polo A. New aspects of Brachytherapy. Journal of Contemporary Brachytherapy. 2011;3(1):45-6.

140. Bose Ribeiro H, Ergonenc HY, Demirel C, Buhler H, Adamietz IA, Galalae R. Short-term outcome of breast-conserving surgery (BCS) and mammosite applicator based interstitial high-dose rate brachytherapy (HDR-BT) in women with breast cancer (BC): Preliminary results from a two-institution collaboration. Strahlentherapie und Onkologie. 2012;188(8):739.

141. Buchwalter G, Brown M. PDEF regulation and role as a survival factor in ER positive breast cancer cells. Cancer research. 2012;72(8 SUPPL. 1):no pagination.

142. Chou J, Lin J, Brenot A, Kim JW, Provot S, Werb Z. GATA3 suppresses metastasis, promotes differentiation and modulates the tumor microenvironment by regulating microRNA-29b expression. Molecular biology of the cell. 2012;23(24):no pagination.

143. Hammer J, Track C, Spiegl KJ, Thames HD, Seewald DH, Labeck W, et al. Breast cancer: External beam radiotherapy and interstitial boost application - An analysis of 1065 patients. Radiotherapy and Oncology. 2012;103:S53.

144. Hannoun-Levi JM, Resch A, Gal J, Niehoff P, Loessl K, Kovacs G, et al. Second conservative treatment for ipsilateral breast tumor recurrence: GEC-ESTRO Breast WG study. Cancer research. 2012;72(24 SUPPL. 3):no pagination.

145. Idoux-Gillet Y, Nassour M, Selmi A, Come C, Deugnier MA, Savagner P. Slug controls p-cadherin and regulates stem/progenitor cell dynamics during mammary gland morphogenesis. European Journal of Cancer. 2012;48:S69-S70.

146. Italiani M, Casale M, Chirico L, Draghini L, Buono E, Muti M, et al. High-dose rate brachytherapy for early breast cancer: Dosimetric data and cosmetic effect. Radiotherapy and Oncology. 2012;103:S129.

147. Jawad MS, Wilkinson JB, Shah C, Gustafson GZ, Fowler A, Mitchell CK, et al. Seven-year clinical outcomes following accelerated partial breast irradiation stratified by astro consensus groupings. International Journal of Radiation Oncology Biology Physics. 2012;84(3 SUPPL. 1):S186.

148. Jonsson P, Strom A, Williams C. Exploring diverging functions of long-term ERbeta expression in two breast cancer cell lines. Endocrine Reviews. 2012;33(3 MeetingAbstracts):no pagination.

149. Keiler LC, Cartwright P, Wennerstrom C, Einstein D. Community hospital experience with accelerated partial breast brachytherapy. International Journal of Radiation Oncology Biology Physics. 2012;84(3 SUPPL. 1):S218.

150. Kenny PA, Chandiramani N. Transgenic expression of a breast-cancer specific GATA3 mutant leads to mammary hyperplasia. Cancer research. 2012;72(24 SUPPL. 3):no pagination.

151. Kung HN, Marks JR, Ashley Chi JT. Glutamine synthetase is a genetic determinant of cell-type specific glutamine independence in breast epithelia. Cancer research. 2012;72(8 SUPPL. 1):no pagination.

152. Lacroix-Triki M, Radosevic-Robin N, Louis B, Roche-Comet I, Soubeyrand MS, Bourgeois H, et al. PT1a, bpN0M0 breast cancer: Clinicopathological characteristics and their impact on treatment decision. Central review of the prospective ODISSEE cohort. Cancer research. 2012;72(24 SUPPL. 3):no pagination.

153. Lin F, Shi J, Wilkerson M, Liu H. Evaluation of GATA3 expression in tumors from various organs. Laboratory Investigation. 2012;92:50A-1A.

154. Liu H, Bockhorn J, Dalton R, Nwachukwu C, Prat A, Yee K, et al. MicroRNAs regulate breast cancer stem cells and spontaneous metastases in orthotopic xenograft models. Cancer research. 2012;72(8 SUPPL. 1):no pagination.

155. Palumbo I, Farneti A, Capezzali G, Falcinelli L, Margaritelli M, Lancellotta V, et al. PBI with interstitial high dose rate brachytherapy: Results of a phase II prospective study. Radiotherapy and Oncology. 2012;103:S247-S8.

156. Peters I, Gebauer K, Hennenlotter J, Kramer M, Abbas M, Merseburger AS, et al. CpG-island methylation of GATA-family members GATA3 and GATA5 in renal cell carcinoma and association with clinicopathological parameters and progression free survival. Urologe - Ausgabe A. 2012;51:107.

157. Pignol J, Doggett S, Crook J, Trombetta M, Caudrelier JM, Fried P, et al. Long term results and ongoing trials of permanent breast seed implant. Radiotherapy and Oncology. 2012;103:S4-S5.

158. Polgar C, Major T, Fodor J, Sulyok Z, Takacsi-Nagy Z, Nemeth G, et al. Breast-conserving therapy with partial or whole breast RT: 10-year results of the Budapest randomized trial. Radiotherapy and Oncology. 2012;103:S35.

159. Taboada Valladares B, Gonzalez Patino E, Salvador Garrido N, Castro Gomez E, Luna Vega V, Calvo Crespo P. Single fraction (7 Gy) of HDR-brachytherapy as boost in breast-conserving therapy: 5 years results in 139 patients. Radiotherapy and Oncology. 2012;103:S127-S8.

160. Vavassori A, Gherardi F, Morra A, Colangione SP, Fodor C, Comi S, et al. HDR brachytherapy for local recurrences after prior breast radiotherapy: Feasibility and preliminary results. Radiotherapy and Oncology. 2012;103:S127.

161. White JR, Winter KA, Kuske RR, Bolton JS, Arthur DW, Scroggins T, et al. Long-term outcome from RTOG 9517: A phase I/II study of accelerated partial breast irradiation (APBI) with mulitcatheter brachytherapy (MCT) following lumpectomy for early-stage breast cancer. Journal of Clinical Oncology. 2012;30(27 SUPPL. 1):no pagination.

162. Yu KD, Zhu R, Zhan M, Shao ZM, Yang W, Symmans WF, et al. Identification of prognosis-relevant subgroups in patients with chemoresistant triple negative breast cancer. Cancer research. 2012;72(24 SUPPL. 3):no pagination.

163. Anderson BM, Das RK, Geye HM, Bradley KA, Patel RR, Kuske RR. Accelerated partial breast irradiation for ductal carcinoma in situ: Single institution experience with median follow-up time of over 5 years. International Journal of Radiation Oncology Biology Physics. 2013;87(2 SUPPL. 1):S238-S9.

164. Anonymous. 17th Meeting of the Radiation Oncology Spanish Society, SEOR 2013. Reports of Practical Oncology and Radiotherapy. 2013;18:no pagination.

165. Arterbery VE, Johnson P. Early clinical results of intraoperative radiation therapy (IORT) during breast conservation using an electronic brachytherapy (EBX) technique. Journal of Clinical Oncology. 2013;31(26 SUPPL. 1):no pagination.

166. Budrukkar A, Pandit P, Jalali R, Gupta S, Parmar V, Upereti R, et al. Impact of adjuvant systemic chemotherapy on wound healing and cosmetic outcome in 224 consecutive patients treated with accelerated partial breast irradiation (APBI) using interstitial brachytherapy. European Journal of Cancer. 2013;49:S455-S6.

167. Cakir A, Ekinci O, Isik Gonul I, Cetin B, Benekli M, Uluoglu O. GATA3 expression and relationship between clinicopathological parameters in invasive breast carcinomas. Laboratory Investigation. 2013;93:32A.

168. Chatterjee S, Bacopulos S, Yang WY, Amemiya Y, Spyropoulos D, Seth A, et al. Loss of IGFBP7 leads to the expansion of luminal progenitors by altering the stromal fibroblasts' ability to support luminal cell differentiation. Cancer research. 2013;73(8 SUPPL. 1):no pagination.

169. Deftereos G, Krishnamurti U, Silverman JF. Gata3 expression in different subtypes of invasive breast carcinoma and comparison with GCDFP15 and mammaglobin. Laboratory Investigation. 2013;93:36A.

170. Desai K, Dendukuri N, Manjunath S, Correa M, Srinath BS, Gopinath KS, et al. A majority of breast cancer specimens with 1-9 % er positivity behave like hormone receptor negative tumors. Annals of Oncology. 2013;24:iii14.

171. Giltnane JM, Balko JM, Wang K, Kuba MG, Mehndi M, Stricker TP, et al. Serial next generation sequencing (NGS) of poor prognosis luminal tumors across treatment history reveals both de novo and acquired alterations potentially associated with endocrine resistance. Cancer research. 2013;73(24 SUPPL. 1):no pagination.

172. Giovanni C, Perou CM, Sander C, Schultz N. The molecular diversity of Luminal A breast cancer. Cancer research. 2013;73(8 SUPPL. 1):no pagination.

173. Jerjees D, Abduljabbar R, Nolan C, Green A, Ellis I, Rakha E. Oestrogen receptor and HER2 differentially activate ERK/MAPK signaling pathway in breast cancer: A large cohort study. Virchows Archiv. 2013;463(2):260.

174. Liao G, Hartmaier RJ, Luthra S, Chandran U, McGuire KP, Puhalla SL, et al. Unique genetic, epigenetic and transcriptomic changes in premenopausal breast cancer suggest novel strategies for therapy. Cancer research. 2013;73(24 SUPPL. 1):no pagination.

175. Liu H, Bockhorn J, Dalton R, Nwachukwu C, Huang S, Prat A, et al. MicroRNA-30c inhibits human breast tumor chemo-resistance by regulating twinfinlin-1 (TWF1) and IL-11. Cancer research. 2013;73(8 SUPPL. 1):no pagination.

176. Markosyan N, Edward C, Ndong V, Smyth E. HER2/neu oncogene induced tumors in mammary epithelial cell COX-2 deficient mice have disease delaying modifications of tumor microenvironment. Cancer research. 2013;73(1 SUPPL. 1):no pagination.

177. Perou CM. The therapeutic implications of genome sequencing and expression analyses for breast cancer. Cancer research. 2013;73(8 SUPPL. 1):no pagination.

178. Pinar Seden o M, Rodriguez Ibarria N, Cabezan Pons M, Rodriguez Melcan J, Gonzalez Machin G, Riveros A, et al. Acelerated partial breast irradiation (APBI) with high dose rate brachytherapy: Feasibility, clinical results in terms of survival, relapse and toxicity. Reports of Practical Oncology and Radiotherapy. 2013;18:S49-S50.

179. Reis-Filho JS. Breast cancer genomics. Journal of Pathology. 2013;229:S3.

180. Strnad V, Ott OJ, Hildebrandt G, Knauerhase H, Potter R, Fietkau R, et al. Phase III multicenter trial: Interstitial brachytherapy alone versus external beam radiation therapy after breast conserving surgery for low-risk invasive carcinoma and low-risk ductal carcinoma in-situ of the female breast-one-year toxicities. Brachytherapy. 2013;12:S15.

181. Wallace M, Chen PY, Wilkinson JB, Fowler A, Jawad MS, Shah C, et al. Fourteen-year outcomes using multicatheter interstitial brachytherapy for accelerated partial breast irradiation. Brachytherapy. 2013;12:S11.

182. Warrick J, Palanisamy N, Siddiqui J, Mehra R, Wu A, Tomlins S, et al. GATA3 expression in nested, micropapillary, and plasmacytoid urothelial carcinomas. Laboratory Investigation. 2013;93:256A.

183. Werb Z. Regulation of metastasis in breast cancer. Cancer research. 2013;73(19 SUPPL. 1):166DUMMY.

184. Aguiar A, Trigo L, Stas N. Accelerated partial breast irradiation (APBI) with multicatheter interstitial brachytherapy over 11 years of practice. Anticancer research. 2014;34(10):6218.

185. Anonymous. 96th Annual Meeting of the American Radium Society, ARS 2014. Oncology. 2014;28(1S):no pagination.

186. Azizi E, Fouladdel S, Deol YS, Bender J, McDermott S, Jiang H, et al. Exploring cancer stem cells heterogeneity via single cell multiplex gene expression analysis. Cancer research. 2014;74(19 SUPPL. 1):no pagination.

187. Brownschidle SS, Mitchell JM, Ambaye AB. GATA-3 immunohistochemistry(IHC) staining pattern in parathyroid and thyroid tissues, using histology and cytology samples. Laboratory Investigation. 2014;94:151A.

188. Gherardi F, Vavassori A, Morra A, Fodor C, Comi S, Cattani F, et al. Partial breast re-irradiation using brachytherapy for local recurrences after prior external beam radiotherapy. Radiotherapy and Oncology. 2014;111:S146-S7.

189. Giltnane JM, Balko J, Mu J, Christiansen J, Murphy D, Mayer I, et al. Genomic alterations associated with resistance to antiestrogens identified by multiplatform molecular analysis in operable ER+ breast cancer. Cancer research. 2014;74(19 SUPPL. 1):no pagination.

190. Gribaudo S, De Sanctis C, Durando A, Katsaros D, Porpiglia M, Richetto V, et al. Ipsilateral Breast Tumour Recurrence (IBTR): Second conservative treatment with Interstitial HDR BRT. Radiotherapy and Oncology. 2014;111:S145.

191. Gupta S, Maitra SK, Ray DS, Saha S, Goswami C, Mukhopadhyay S. Long term analysis of cosmetic outcome after breast conservation and correlation with type of radiotherapy boost - an Indian experience. European Journal of Cancer. 2014;50:S218.

192. Hepel JT, Leonard KL, Sha S, Hiatt JR, Graves TA, Wiggins DL, et al. Non-invasive image-guided breast brachytherapy (NIBB) to deliver accelerated partial breast irradiation (APBI): Analysis of acute toxicity and early outcomes. International Journal of Radiation Oncology Biology Physics. 2014;90(1 SUPPL. 1):S136.

193. Hosseini N, Ghorab Z, Keith J, Slodkowska E, Lu FI, Han G, et al. Expression of GATA3 in brain metastases of breast origin. Laboratory Investigation. 2014;94:437A.

194. Jerjees D, Nejm O, Mirza S, Abduljabbar R, Green A, Tighe P, et al. The biological and clinical significance of phosphorylated mTOR (pi3k/AKt pathway related) and its association with estrogen receptor and HER2 pathways. Virchows Archiv. 2014;465(1 SUPPL. 1):S99.

195. Liu H. Epigenetic characterization and specific targeting of breast cancer stem cells. Cancer research. 2014;74(19 SUPPL. 1):no pagination.

196. McCleskey BC, Nguyen T, Grizzle WE, Zhang K, Hameed O, Siegal GP, et al. GATA3 has prognostic value in advanced breast cancer. Laboratory Investigation. 2014;94:67A.

197. Pinar B, Blanco J, Riveros-Perez A, Garcia-Cabrera L, Cabezon-Pons MA, Rodriguez-Ibarria N, et al. Acelerated Partial Breast Irradiation (APBI) with high dose rate brachytherapy in luminal breast cancer. Radiotherapy and Oncology. 2014;111:S61.

198. Polgar C, Major T, Sulyok Z, Takacsi-Nagy Z, Fodor J. Toxicity and cosmetic results of partial vs whole breast irradiation: 10-year results of a randomized trial. Radiotherapy and Oncology. 2014;111:S60.

199. Polgar C, Major T, Sulyok Z, Takacsi-Nagy Z, Fodor J. Long-term toxicity and cosmetic results of partial versus whole breast irradiation: 10-year results of a phase iii APBI trial. International Journal of Radiation Oncology Biology Physics. 2014;90(1 SUPPL. 1):S133-S4.

200. Polo Rubio J. Evidence for a dose-response relationship in breast cancer. Radiotherapy and Oncology. 2014;111:S85-S6.

201. Saha S, Ghosh Dastidar A, Das Gupta P, Chattopadhyay S, Ghosh K. Comparison between graphical optimization and geometrical optimization in HDR interstial breast implants. Radiotherapy and Oncology. 2014;111:S330-S1.

202. Sarkar S, Saha S, Mitra A. Long term analysis of cosmetic outcome in breast conservation and its correlation with type of radiotherapy boost-an Indian experience. International Journal of Gynecological Cancer. 2014;24(9 SUPPL. 4):1037.

203. Schuster JM, Chipko C, Saraiya S, Hamid S, Sha S, Jolly M, et al. Update on a multi-institutional study of noninvasive breast brachytherapy for tumor bed boost: Cosmesis and tumor control. Brachytherapy. 2014;13:S19.

204. Shahi P, Slorach EM, Chou J, Lawson D, Yu Y, Werb Z. Zpo2 promotes aggressive breast cancer development through downregulation of GATA3. Cancer research. 2014;74(19 SUPPL. 1):no pagination.

205. Singhal H, Greene G. Genome-wide crosstalk of estrogen and progesterone signaling in breast cancer. Cancer research. 2014;74(19 SUPPL. 1):no pagination.

206. Sunny J, Irfan B, Anshul B, Anil T, Kundan CS. A rare incidence of synchronous endometrial and breast carcinoma treated successfully at a tertiary care center: A report with review of literature. Journal of Cancer Research and Therapeutics. 2014;10:S49-S50.

207. Takaku M, Adomas A, Grimm SA, Takashi S, Wade PA. GATA3 mutations in breast cancer. Cancer research. 2014;74(19 SUPPL. 1):no pagination.

208. Tilson MP, Illei PB, Argani P, Taube JM, Cimino-Mathews A. The utility of GATA3 in distinguishing breast ductal carcinoma from skin adnexal neoplasms. Laboratory Investigation. 2014;94:143A-4A.

209. Young RB, Medbery ICA, Young MM, Morrison AE. Accelerated partial breast irradiation with multicatheter high-dose-rate brachytherapy: Feasibility and results in a private practice cohort. Brachytherapy. 2014;13:S83-S4.

210. Aguiar A, Trigo L, Stas N. APBI single-centre experience over a decade-risk estimates and indication variations within current guidelines. Radiotherapy and Oncology. 2015;115:S212-S3.

211. Azim HA, Nguyen B, Brohee S, Piccart-Gebhart MJ, Sotiriou C. The pattern of somatic mutations and chromosomal copy number variations (CNV) in young breast cancer (BC) patients (pts). Journal of Clinical Oncology. 2015;33(15 SUPPL. 1):no pagination.

212. Chichel A, Skowronek J. Thermal boost combined with HDR brachytherapy in breast conserving therapy-a study update after 7-year follow-up. Brachytherapy. 2015;14:S40.

213. Ciriello G, Gatza ML, Hoadley KA, Zhang H, Rhie SK, Bowlby R, et al. Comprehensive molecular characterization of invasive lobular breast tumors. Cancer research. 2015;75(9 SUPPL. 1):no pagination.

214. Desmedt C, Gundem G, Zoppoli G, Pruneri G, Biganzoli E, Fornili M, et al. Characterization and clinical relevance of the genomic alterations defining lobular breast cancer. Cancer research. 2015;75(9 SUPPL. 1):no pagination.

215. Fountzilas G, Dimopoulos MA, Kouvatseas G, Timotheadou E, Pentheroudakis GE, Gogas H, et al. Effect of mutant TP53 genotypes on the outcome of breast cancer (BC) patients in different clinical tumor subtypes. Journal of Clinical Oncology. 2015;33(15 SUPPL. 1):no pagination.

216. Gaudet M, Trudel-Sabourin J, Nguyen J, Wright D, Doiron A, Pharand-Charbonneau M, et al. Long-term results of interstitial brachytherapy for accelerated partial-breast irradiation in a community center. Brachytherapy. 2015;14:S105.

217. Gillard C, Chand ME, Gal J, Lamchamkee D, Gautier M, Raoust I, et al. Ipsilateral breast tumor recurrence: 5-year clinical outcome after salvage lumpectomy and interstitial brachytherapy. International Journal of Radiation Oncology Biology Physics. 2015;93(3 SUPPL. 1):S225.

218. Griffith OL, Griffith M, Luo J, Hundall J, Miller CA, Larson DE, et al. Prognostic effects of gene mutation in estrogen receptor positive breast cancer. Cancer research. 2015;75(9 SUPPL. 1):no pagination.

219. Gyorffy B, Pongor L, Kormos M. Linking genotype to clinical outcome in breast cancer by combining NGS and gene chip data. Cancer research. 2015;75(9 SUPPL. 1):no pagination.

220. Kamrava M, Kuske RR, Chen P, Hayes J, Anderson B, Quiet C, et al. Outcomes of node positive patients treated with accelerated partial breast irradiation via interstitial multicatheter brachytherapy: The pooled registry of multicatheter interstitial sites (promis) experience. Brachytherapy. 2015;14:S19.

221. Kotoula V, Lakis S, Alexopoulou Z, Charalambous E, Papadopoulou K, Lyberopoulou A, et al. Clinical relevance of TP53 mutations and genomic instability in node positive breast cancer. Cancer research. 2015;75(9 SUPPL. 1):no pagination.

222. Kovacs G. Focal therapy alone in primary and recurrent disease. Radiotherapy and Oncology. 2015;115:S114.

223. Lancellotta V, Chirico L, Palumbo I, Anselmo P, Zucchetti C, Italiani M, et al. PBI with interstitial high-dose-rate brachytherapy: Results of a phase II prospective study. Radiotherapy and Oncology. 2015;115:S19-S20.

224. Liu H. Acquired differentiation and loss of malignancy of pulmonary metastases in breast cancer. Cancer research. 2015;75(15 SUPPL. 1):no pagination.

225. Mann JM, Osian AD, Brandmaier A, Yan W, Wu G, Christos P, et al. Excellent long-term breast preservation rate following accelerated partial-breast irradiation using a balloon device. International Journal of Radiation Oncology Biology Physics. 2015;93(3 SUPPL. 1):E34.

226. Martinelli P, Sainz B, Carrillo-De Santa Pau E, Rinaldi L, Del Pozo N, Real FX. GATA 3/6 control tissue-specific epithelial programmes and a basal-like phenotype in multiple cancer types, including PDAC. Pancreatology. 2015;15(3 SUPPL. 1):S30.

227. McMullen E, Samuelson M, Bellizzi A. GATA3 is frequently expressed by estrogen receptor-negative breast cancers: A comparison of two commercially available monoclonal antibodies. Laboratory Investigation. 2015;95:56A.

228. Pathak RS, Mahantshetty U, Kinhikar R, Jalali R, Parmar V, Budrukkar A, et al. Long term results of a prospective study of internal mammary chain (IMC) brachytherapy. Journal of Clinical Oncology. 2015;33(15 SUPPL. 1):no pagination.

229. Rdguez-Ibarria N, Pinar B, Cabezon MA, Rodriguez Melcon N, Blanco J, Garcia-Cabrera L, et al. Risk adapted interstitial brachytherapy for breast cancer treatment: Analysis of a single institution results. Radiotherapy and Oncology. 2015;115:S19.

230. Ristevska-Dimitrovska G, Stefanovski P, Smichkoska S, Dejanova B. Effects of adjuvant endocrine therapy on depression and quality of life in early breast cancer patients. European Neuropsychopharmacology. 2015;25:S403.

231. Saha S, Dastidar AG, Gupta S. Long term analysis of cosmetic outcome of breast conservation and correlation with electron Vs. HDR boost-an Indian experience. Brachytherapy. 2015;14:S40.

232. Schuster JM, Chipko C, Quiet CA, Benda RK, Sha SJ, Kuruvilla AM, et al. Noninvasive breast brachytherapy boost: Cosmesis and tumor control. International Journal of Radiation Oncology Biology Physics. 2015;93(3 SUPPL. 1):E35-E6.

233. Smith LA, Kuske RR, Nelums E, Sadeghi A. Plesiotherapy boost after breast reconstruction following mastectomy. International Journal of Radiation Oncology Biology Physics. 2015;93(3 SUPPL. 1):E55.

234. Takaku M, Grimm SA, Shimbo T, Perera L, Machida S, Kurumizaka H, et al. GATA3 modulates chromatin structure to establish active enhancers in breast cancer cells. Cancer research. 2015;75(15 SUPPL. 1):no pagination.

235. Amendola B, Perez N, Suarez JB, Amendola M, Wu X. Accelerated partial breast irradiation (APBI) with balloon brachytherapy in early breast cancer: 12 year experience in 285 patients treated in a single institution. Brachytherapy. 2016;15:S42.

236. Balko JM, Hicks M, Berger MF, Solit DB, Bouvier N, Sanders ME, et al. Genomic alterations indicative of a luminal A subtype associate with clinical benefit to buparlisib and letrozole in endocrine-resistant ER+/HER2- metastatic breast cancer. Cancer research. 2016;76(4 SUPPL. 1):no pagination.

237. Benz SC, Rabizadeh S, Cecchi F, Beckman MW, Brucker SY, Hartmann A, et al. Integrating whole genome sequencing data with RNAseq, pathway analysis, and quantitative proteomics to determine prognosis after standard adjuvant treatment with trastuzumab and chemotherapy in primary breast cancer patients. Cancer research. 2016;76(4 SUPPL. 1):no pagination.

238. Martens JWM, Smid M, Rodriguez-Gonzalez G, Sieuwerts AM, Prager-Van Der Smissen WJC, Van Der Vlugt-Daane M, et al. Mutational signatures impact the breast cancer transcriptome and distinguish mitotic from immune response pathways. Cancer research. 2016;76(4 SUPPL. 1):no pagination.

239. Meszaros N, Major T, Stelczer G, Smanyko V, Matrai T, Polgar C. >5-years results of second breast conserving therapy (BCT) with high-dose-rate (HDR) interstitial brachytherapy (iBT): A single institutional experience. Brachytherapy. 2016;15:S44.

240. Polgar C, Strnad V, Ott OJ, Hildebrandt G, Kauer-Dorner D, Knauerhase H, et al. Incidences of late toxicities and cosmetic results after accelerated partial breast irradiation with multi-catheter brachytherapy or whole Breast Irradiation: 5-year results of the GEC-ESTRO Phase III APBI trial. Brachytherapy. 2016;15:S47.

241. Rodriguez-Ibarria N, Pinar B, Cabezon MA, Lloret M, Lara PC. Interstitial high-dose rate (HDR) brachytherapy: A risk adapted treatment for breast cancer. Brachytherapy. 2016;15:S204.

242. Sfeir A. The role of mammalian polymerase theta in DNA repair. FASEB Journal. 2016;30:no pagination.

243. Tanaka K, Tokunaga E, Inoue Y, Ueo H, Yamashita N, Sagara Y, et al. The relationship between the expression of FOXA1 and GATA3 and the efficacy of neoadjuvant endocrine therapy. Cancer research. 2016;76(4 SUPPL. 1):no pagination.

244. Polgar C, Sulyok Z, Major T, Riedl E, Somogyi A, Fodor J, et al. Reexcision and perioperative brachytherapy in the treatment of local relapse after breast conservation: a possible alternative to mastectomy. Magyar sebeszet. 2000;53(3):120-3.

245. Calvo FA, Meirino RM, Orecchia R. Intraoperative radiation therapy - Part 2. Clinical results. Critical Reviews in Oncology Hematology. 2006;59(2):116-27. doi: 10.1016/j.critrevonc.2006.04.004. PubMed PMID: WOS:000239884200004.

246. Fang SH, Chen Y, Weigel RJ. GATA-3 as a marker of hormone response in breast cancer. The Journal of surgical research. 2009;157(2):290-5. Epub 2008/12/09. doi: 10.1016/j.jss.2008.07.015. PubMed PMID: 19059610.

247. Habashy HO, Powe DG, Ball G, Glaab E, Soria D, Garibaldi J, et al. Luminal-like oestrogen receptor-positive breast cancer: Identification of prognostic biological subclasses. European Journal of Cancer, Supplement. 2010;8(3):91.

248. Bahadur YA, Constantinescu CT. Tumor bed boost radiotherapy in breast cancer: A review of current techniques. Saudi medical journal. 2012;33(4):353-66.

249. Debenham BJ, Hu KS, Harrison LB. Present status and future directions of intraoperative radiotherapy. Lancet Oncology. 2013;14(11):E457-E64. PubMed PMID: WOS:000325091900021.

250. Ordonez NG. Value of GATA3 immunostaining in tumor diagnosis: a review. Advances in anatomic pathology. 2013;20(5):352-60. Epub 2013/08/14. doi: 10.1097/PAP.0b013e3182a28a68. PubMed PMID: 23939152.

251. Previati M, Manfrini M, Galasso M, Zerbinati C, Palatini J, Gasparini P, et al. Next generation analysis of breast cancer genomes for precision medicine. Cancer letters. 2013;339(1):1-7.

252. Kirisits C, Rivard MJ, Baltas D, Ballester F, De Brabandere M, van der Laarse R, et al. Review of clinical brachytherapy uncertainties: Analysis guidelines of GEC-ESTRO and the AAPM. Radiotherapy and Oncology. 2014;110(1):199-212. doi: 10.1016/j.radonc.2013.11.002. PubMed PMID: WOS:000333792100031.

253. Siddiqui MT, Seydafkan S, Cohen C. GATA3 Expression in Metastatic Urothelial Carcinoma in Fine Needle Aspiration Cell Blocks: A Review of 25 Cases. Diagnostic cytopathology. 2014;42(9):809-15. doi: 10.1002/dc.23131. PubMed PMID: WOS:000339570000014.

254. Baxter C, Donnellan P. HER2 positive metastatic adenocarcinoma of skin: A case report. Irish Journal of Medical Science. 2015;184(7 SUPPL. 1):S300.

255. Kim S. New and emerging factors in tumorigenesis: An overview. Cancer Management and Research. 2015;7:225-39.

256. Zardavas D, Irrthum A, Swanton C, Piccart M. Clinical management of breast cancer heterogeneity. Nature Reviews Clinical Oncology. 2015;12(7):381-94.

257. Asch-Kendrick R, Cimino-Mathews A. The role of GATA3 in breast carcinomas: A review. Human pathology. 2016;48:37-47.

258. Hammer J, Seewald DH, Track C, Zoidl JP, Labeck W. Breast cancer: Primary treatment with external-beam radiation therapy and high-dose-rate iridium implantation. Radiology. 1994;193(2):573-7.

259. Track C, Seewald DH, Zoidl JP, Hammer J. External radiation and HDR-brachytherapy in the treatment of breast cancer. Strahlentherapie und Onkologie. 1994;170(4):213-7.

260. Lee DJ, Mayer R, Hallinan L. Outpatient interstitial thermoradiotherapy. Cancer. 1996;77(11):2363-70.

261. Aragona M, Muscatello MRA, Mesiti M. Depressive mood disorders in patients with operable breast cancer. Journal of Experimental & Clinical Cancer Research. 1997;16(1):111-8. PubMed PMID: WOS:A1997WX28100019.

262. Hammer J, Track C, Seewald DH, Zoidl JP, Labeck W, Putz E, et al. Breast cancer: external beam radiotherapy and interstitial iridium implantation - 10-year clinical results. Progress in Radio-Oncology Vi1998. p. 863-71.

263. Mazeron JJ, Gerbaulet A, Simon JM, Hardiman C. How to optimize therapeutic ratio in brachytherapy of head and neck squamous cell carcinoma? Acta Oncologica. 1998;37(6):583-91.

264. De Pree C, Popowski Y, Weber D, Nouet P, Rouzaud M, Kurtz JM. Feasibility and tolerance of pulsed dose rate interstitial brachytherapy. International Journal of Radiation Oncology Biology Physics. 1999;43(5):971-6.

265. Dubben HH, Beck-Bornholdt HP. Actuarial analysis of time-failure data and its relevance for interpretation of results. Audit of the Journal "Strahlentherapie und onkologie". Strahlentherapie Und Onkologie. 2000;176(12):547-+. doi: 10.1007/PL00002323. PubMed PMID: WOS:000165775600001.

266. Manning PA, Arthur DW, Schmidt-Ullrich RK, Arnfield MR, Amir C, Zwicker RD. Interstitial high-dose-rate brachytherapy boost: The feasibility and cosmetic outcome of a fractionated outpatient delivery scheme. International Journal of Radiation Oncology Biology Physics. 2000;48(5):1301-6. PubMed PMID: WOS:000165604600004.

267. Hammer J, Track C, Seewald DH. Breast cancer: The medial tumour location - An unfavourable disease! - Results from 645 patients (1984-1995). Progress in Radio-Oncology Vii, Proceedings2001. p. 493-8.

268. Harms W, Becker HD, Krempien R, Wannenmacher M. Contemporary role of modern brachytherapy techniques in the management of malignant thoracic tumors. Seminars in surgical oncology. 2001;20(1):57-65. doi: 10.1002/ssu.1017. PubMed PMID: WOS:000167906600008.

269. Kolotas C, Zamboglou N. Role of interstitial brachytherapy in the treatment of malignant disease. Onkologie. 2001;24(3):222-8. Epub 2001/07/17. doi: 55084. PubMed PMID: 11455214.

270. Polgar C, Fodor J, Orosz Z, Major T, Takacsi-Nagy Z, Mangel LC, et al. Electron and high-dose-rate brachytherapy boost in the conservative treatment of stage I-II breast cancer - First results of the randomized Budapest boost trial. Strahlentherapie Und Onkologie. 2002;178(11):615-23. doi: 10.1007/s00066-002-1053-1. PubMed PMID: WOS:000179403500005.

271. Stranzl H, Gabor S, Mayer R, Prettenhofer U, Wurzinger G, Hackl A. Fractionated intraluminal HDR 192Ir brachytherapy as palliative treatment in patients with endobronchial metastases from non-bronchogenic primaries. Strahlentherapie und Onkologie. 2002;178(8):442-5.

272. Strauss HG, Kuhnt T, Laban C, Puschmann D, Pigorsch S, Dunst J, et al. Chemoradiation in cervical cancer with cisplatin and high-dose rate brachytherapy combined with external beam radiotherapy: Results of a phase-II study. Strahlentherapie und Onkologie. 2002;178(7):378-85.

273. Yoshida K, Nose T, Koizumi M, Mitomo M, Nishiyama K, Yoshida M. The usefulness of metal markers for CTV-based dose prescription in high-dose-rate interstitial brachytherapy. Journal of JASTRO. 2002;14(4):253-60.

274. Arthur DW, Koo D, Zwicker RD, Tong SD, Bear HD, Kaplan BJ, et al. Partial breast brachytherapy after lumpectomy: Low-dose-rate and high-dose-rate experience. International Journal of Radiation Oncology Biology Physics. 2003;56(3):681-9. doi: 10.1016/S0360-3016(03)00120-2. PubMed PMID: WOS:000183361800011.

275. Carlson DJ, Stewart RD, Li XA, Jennings K, Wang JZ, Guerrero M. Comparison of in vitro and in vivo alpha/beta ratios for prostate cancer. Physics in Medicine and Biology. 2004;49(19):4477-91. doi: 10.1088/0031-9155/49/19/003. PubMed PMID: WOS:000224663300003.

276. Dickler A, Kirk M, Choo J, Hsi WC, Chu J, Dowlatshahi K, et al. Treatment volume and dose optimization of MammoSite breast brachytherapy applicator. International Journal of Radiation Oncology Biology Physics. 2004;59(2):469-74.

277. Miller DV, Leontovich AA, Lingle WL, Suman VJ, Mertens ML, Lillie J, et al. Utilizing Nottingham Prognostic Index in microarray gene expression profiling of breast carcinomas. Modern pathology : an official journal of the United States and Canadian Academy of Pathology, Inc. 2004;17(7):756-64. Epub 2004/04/10. doi: 10.1038/modpathol.3800114. PubMed PMID: 15073601.

278. Ott OJ, Potter R, Hammer J, Hildebrandt G, Lotter M, Resch A, et al. Accelerated partial breast irradiation with iridium-192 multicatheter PDR/HDR brachytherapy - Preliminary results of the German-Austrian multicenter trial. Strahlentherapie Und Onkologie. 2004;180(10):642-9. doi: 10.1007/s00066-004-1294-2. PubMed PMID: WOS:000224523600006.

279. Taschereau R, Stauffer PR, Hsu IC, Schlorff JL, Milligan AJ, Pouliot J. Radiation dosimetry of a conformal heat-brachytherapy applicator. Technology in Cancer Research & Treatment. 2004;3(4):347-58. PubMed PMID: WOS:000223460500004.

280. von Tempelhoff GF, Heilmann L, Pollow K, Hommel G. Monitoring of rheologic variables during postoperative high-dose brachytherapy for uterine cancer. Clinical and Applied Thrombosis-Hemostasis. 2004;10(3):239-48. doi: 10.1177/107602960401000306. PubMed PMID: WOS:000222942700006.

281. Beato Tortajada I, Guinot Rodriguez JL, Arribas Alpuente L, Aguayo Martos M, Carrascosa Perez M, Tortajada Azcutia M, et al. Single fraction boost with high dose rate interstitial brachytherapy in conservative treatment of breast carcinoma. Clinical & translational oncology : official publication of the Federation of Spanish Oncology Societies and of the National Cancer Institute of Mexico. 2005;7(9):404-8.

282. Kinhikar RA, Deshpande SS, Mahantshetty U, Sarin R, Shrivastava SK, Deshpande DD. HDR brachytherapy combined with 3-D conformal vs. IMRT in left-sided breast cancer patients including internal mammary chain: comparative analysis of dosimetric and technical parameters. Journal of applied clinical medical physics / American College of Medical Physics. 2005;6(3):1-12.

283. Li J, Huang L, Zhang X. Radiation injury and cosmetic results of breast conserving treatment. Chinese Journal of Clinical Oncology. 2005;32(13):742-4.

284. Srinivas SK, Reddy KS, Vivekanandam S, Parthasarathy V. Role of template guided interstitial implants in breast conservation therapy. Journal of Cancer Research and Therapeutics. 2005;1(2):79-83.

285. Wronczewska A, Makarewicz R, Kabacinska R, Zuchora A. Does interstitial HDR brachytherapy for breast cancer increase soft tissue fibrosis? Reports of Practical Oncology and Radiotherapy. 2005;10(3):119-23.

286. Chen PY, Vicini FA, Benitez P, Kestin LL, Wallace M, Mitchell C, et al. Long-term cosmetic results and toxicity after accelerated partial-breast irradiation - A method of radiation delivery by interstitial Brachytherapy for the treatment of early-stage breast carcinoma. Cancer. 2006;106(5):991-9. doi: 10.1002/cncr.21681. PubMed PMID: WOS:000235822700003.

287. Neumanova R, Petera J, Kuricka R, Macharova H, Dvorak K, Pacovsky Z, et al. The role of interstitial HDR brachytherapy boost in the treatment of early breast cancer. Klinicka Onkologie. 2006;19(1):4-8.

288. Niehoff P, Dietrich J, Ostertag H, Schmid A, Kohr P, Kimmig B, et al. High-dose-rate (HDR) or pulsed-dose-rate (PDR) peri-operative interstitial intensity-modulated brachytherapy (IMBT) for local recurrences of previously irradiated breast or thoracic wall following breast cancer. Strahlentherapie Und Onkologie. 2006;182(2):102-+. doi: 10.1007/s00066-006-1496-x. PubMed PMID: WOS:000235430300007.

289. Terlikiewicz J, Makarewicz R. The role of HDR brachytherapy in palliative treatment of patients with advanced cancer. Polska Medycyna Paliatywna. 2006;5(2):62-70.

290. Badve S, Turbin D, Thorat MA, Morimiya A, Nielsen TO, Perou CM, et al. FOXA1 expression in breast cancer - Correlation with luminal subtype A and survival. Clinical Cancer Research. 2007;13(15):4415-21. doi: 10.1158/1078-0432.CCR-07-0122. PubMed PMID: WOS:000248525100017.

291. Garvin JT, McNeill RE, Hennessy E, Miller N, Kerin MJ. Gata3 and Bag1 over-Expression Are Associated with Good Prognosis in Node Negative Breast Cancer. Irish Journal of Medical Science. 2007;176:S20-S. PubMed PMID: WOS:000207618400020.

292. Mignogna M, Marconi A, Francesconi D, Lorenzini E, Quattrocchi M, Ducci F. Partial breast irradiation with interstitial high dose-rate brachytherapy: Acute and late toxicities & cosmetic results. Nowotwory. 2007;57(6):685-6.

293. Ott OJ, Lotter M, Sauer R, Strnad V. Accelerated partial-breast irradiation with interstitial implants - The clinical relevance of the calculation of skin doses. Strahlentherapie Und Onkologie. 2007;183(8):426-31. doi: 10.1007/s00066-007-1738-6. PubMed PMID: WOS:000248733500004.

294. Roper B, Astner ST, Heydemann-Obradovic A, Thamm R, Jacob V, Holzel D, et al. Ten-year data on 138 patients with endometrial carcinoma and postoperative vaginal brachytherapy alone: No need for external-beam radiotherapy in low and intermediate risk patients. Gynecologic oncology. 2007;107(3):541-8.

295. Soumarova R, Homola L, Perkova H, Czudek S, Skrovina M, Adamcik L. [The role of interstitial brachytherapy in multimodality management of solid tumors]. Rozhledy v chirurgii : mesicnik Ceskoslovenske chirurgicke spolecnosti. 2007;86(10):533-9. Epub 2007/12/11. PubMed PMID: 18064791.

296. Bouras T, Pal B, Vaillant F, Harburg G, Asselin-Labat ML, Oakes SR, et al. Notch Signaling Regulates Mammary Stem Cell Function and Luminal Cell-Fate Commitment. Cell Stem Cell. 2008;3(4):429-41. doi: 10.1016/j.stem.2008.08.001. PubMed PMID: WOS:000260149800013.

297. Jereczek-Fossa BA, Kowalczyk A, D'Onofrio A, Catalano G, Garibaldi C, Boboc G, et al. Three-dimensional conformal or stereotactic reirradiation of recurrent, metastatic or new primary tumors. Strahlentherapie Und Onkologie. 2008;184(1):36-40. doi: 10.1007/s00066-008-1783-9. PubMed PMID: WOS:000252282600006.

298. Polo A. Pulsed dose rate brachytherapy. Clinical and Translational Oncology. 2008;10(6):324-33.

299. Badve S, Nakshatri H. Oestrogen-receptor-positive breast cancer: towards bridging histopathological and molecular classifications. Journal of clinical pathology. 2009;62(1):6-12. Epub 2008/09/17. doi: 10.1136/jcp.2008.059899. PubMed PMID: 18794199.

300. Buonomo SBC, Wu YP, Ferguson D, de Lange T. Mammalian Rif1 contributes to replication stress survival and homology-directed repair. Journal of Cell Biology. 2009;187(3):385-98. doi: 10.1083/jcb.200902039. PubMed PMID: WOS:000271374200010.

301. Chadha M, Mehta P, Feldman S, Boolbol SK, Harrison LB. Intraoperative High-Dose-Rate Brachytherapy-A Novel Technique in the Surgical Management of Axillary Recurrence. Breast Journal. 2009;15(2):140-5. doi: 10.1111/j.1524-4741.2009.00688.x. PubMed PMID: WOS:000264087900004.

302. Deblois G, Hall JA, Perry MC, Laganiere J, Ghahrermani M, Park M, et al. Genome-Wide Identification of Direct Target Genes Implicates Estrogen-Related Receptor alpha as a Determinant of Breast Cancer Heterogeneity. Cancer research. 2009;69(15):6149-57. doi: 10.1158/0008-5472.CAN-09-1251. PubMed PMID: WOS:000268737900019.

303. Emerson JW, Dolled-Filhart M, Harris L, Rimm DL, Tuck DP. Quantitative assessment of tissue biomarkers and construction of a model to predict outcome in breast cancer using multiple imputation. Cancer Informatics. 2009;7:29-40.

304. Hennessy BT, Gonzalez-Angulo AM, Stemke-Hale K, Gilcrease MZ, Krishnamurthy S, Lee JS, et al. Characterization of a Naturally Occurring Breast Cancer Subset Enriched in Epithelial-to-Mesenchymal Transition and Stem Cell Characteristics. Cancer research. 2009;69(10):4116-24. doi: 10.1158/0008-5472.CAN-08-3441. PubMed PMID: WOS:000266214400006.

305. Lazure KE, Lydiatt WM, Denman D, Burke WJ. Association between Depression and Survival or Disease Recurrence in Patients with Head and Neck Cancer Enrolled in a Depression Prevention Trial. Head and Neck-Journal for the Sciences and Specialties of the Head and Neck. 2009;31(7):888-92. doi: 10.1002/hed.21046. PubMed PMID: WOS:000267605200007.

306. Matsuura K, Yamaguchi Y, Osaki A, Ohara M, Okita R, Emi A, et al. FOXP3 expression of micrometastasis-positive sentinel nodes in breast cancer patients. Oncology reports. 2009;22(5):1181-7. Epub 2009/09/30. PubMed PMID: 19787238.

307. Ott OJ, Lotter M, Fietkau R, Strnad V. Accelerated partial-breast irradiation with interstitial implants : AAnalysis of factors affecting cosmetic outcome. Strahlentherapie und Onkologie. 2009;185(3):170-6.

308. Ott OJ, Lotter M, Fietkau R, Strnad V. Accelerated Partial-Breast Irradiation with Interstitial Implants. Strahlentherapie Und Onkologie. 2009;185(3):170-6. doi: 10.1007/s00066-009-1943-6. PubMed PMID: WOS:000264699500005.

309. Wadasadawala T, Sarin R, Budrukkar A, Jalali R, Munshi A, Badwe R. Accelerated partial-breast irradiation v conventional whole-breast radiotherapy in early breast cancer: A case-control study of disease control, cosmesis, and complications. Journal of Cancer Research and Therapeutics. 2009;5(2):93-101. doi: 10.4103/0973-1482.52794. PubMed PMID: WOS:000267145100006.

310. Adrien D, Ludger KH, Ingo S, Oliver M, Cornelius K, Michael CA, et al. Malignant fibrous histiocytoma-pleomorphic sarcoma, NOS gene expression, histology, and clinical course. A pilot study. Langenbecks Archives of Surgery. 2010;395(3):261-75. doi: 10.1007/s00423-009-0465-0. PubMed PMID: WOS:000275749400012.

311. Chen JQ, Litton J, Xiao L, Zhang HZ, Warneke CL, Wu Y, et al. Quantitative immunohistochemical analysis and prognostic significance of TRPS-1, a new GATA transcription factor family member, in breast cancer. Hormones & cancer. 2010;1(1):21-33. Epub 2010/02/01. doi: 10.1007/s12672-010-0008-8. PubMed PMID: 21761348.

312. Csaba P, Major T, Janos F, Sulyok Z, Andras S, Katalin L, et al. Accelerated partial-breast irradiation using high-dose-rate interstitial brachytherapy: 12-year update of a prospective clinical study. Radiotherapy and Oncology. 2010;94(3):274-9. doi: 10.1016/j.radonc.2010.01.019. PubMed PMID: WOS:000276619500003.

313. Guedea F, Venselaar J, Hoskin P, Hellebust TP, Peiffert D, Londres B, et al. Patterns of care for brachytherapy in Europe: Updated results. Radiotherapy and Oncology. 2010;97(3):514-20.

314. Ivyna Bong PN, Zakaria Z, Muhammad R, Abdullah N, Ibrahim N, Emran NA, et al. Expression and mutational analysis of GATA3 in Malaysian breast carcinomas. Malaysian Journal of Pathology. 2010;32(2):117-22.

315. McCune K, Bhat-Nakshatri P, Thorat MA, Nephew KP, Badve S, Nakshatri H. Prognosis of hormone-dependent breast cancers: implications of the presence of dysfunctional transcriptional networks activated by insulin via the immune transcription factor T-bet. Cancer research. 2010;70(2):685-96. Epub 2010/01/14. doi: 10.1158/0008-5472.can-09-1530. PubMed PMID: 20068169; PubMed Central PMCID: PMCPMC2807987.

316. McCune K, Mehta R, Thorat MA, Badve S, Nakshatri H. Loss of ERalpha and FOXA1 expression in a progression model of luminal type breast cancer: insights from PyMT transgenic mouse model. Oncology reports. 2010;24(5):1233-9. Epub 2010/09/30. PubMed PMID: 20878115; PubMed Central PMCID: PMCPMC2948410.

317. Polgar C, Major T, Fodor J, Sulyok Z, Somogyi A, Lovey K, et al. Accelerated partial-breast irradiation using high-dose-rate interstitial brachytherapy: 12-year update of a prospective clinical study. Radiotherapy and Oncology. 2010;94(3):274-9.

318. Shi CY, Guo BQ, Cheng CY, Eng T, Papanikolaou N. Applications of tissue heterogeneity corrections and biologically effective dose volume histograms in assessing the doses for accelerated partial breast irradiation using an electronic brachytherapy source. Physics in Medicine and Biology. 2010;55(18):5283-97. doi: 10.1088/0031-9155/55/18/003. PubMed PMID: WOS:000281481500003.

319. Yeo SG, Kim J, Kwak GH, Kim JY, Park K, Kim ES, et al. Accelerated partial breast irradiation using multicatheter brachytherapy for select early-stage breast cancer: local control and toxicity. Radiation Oncology. 2010;5. doi: 10.1186/1748-717X-5-56. PubMed PMID: WOS:000280269000001.

320. Chen JQ, Bao Y, Litton J, Xiao L, Zhang HZ, Warneke CL, et al. Expression and relevance of TRPS-1: a new GATA transcription factor in breast cancer. Hormones & cancer. 2011;2(2):132-43. Epub 2011/07/16. doi: 10.1007/s12672-011-0067-5. PubMed PMID: 21761336.

321. Chen YZ, Chen CM, Yang BL, Xu QH, Wu F, Liu F, et al. Estrogen receptor-related genes as an important panel of predictors for breast cancer response to neoadjuvant chemotherapy. Cancer letters. 2011;302(1):63-8. doi: 10.1016/j.canlet.2010.12.014. PubMed PMID: WOS:000287614600008.

322. Jain RK, Mehta RJ, Nakshatri H, Idrees MT, Badve SS. High-level expression of forkhead-box protein A1 in metastatic prostate cancer. Histopathology. 2011;58(5):766-72. doi: 10.1111/j.1365-2559.2011.03796.x. PubMed PMID: WOS:000289465400013.

323. Lakosi F, Antal G, Vandulek C, Kovacs A, Toller GL, Rakasz I, et al. Open MR-Guided High-Dose-Rate (HDR) Prostate Brachytherapy: Feasibility and Initial Experiences Open MR-Guided High-Dose-Rate (HDR) Prostate Brachytherapy. Pathology & Oncology Research. 2011;17(2):315-24. doi: 10.1007/s12253-010-9319-x. PubMed PMID: WOS:000290587300018.

324. Sato K, Takayanagi H, Mizuno Y, Shimo T, Kato M. Intraoperative open-cavity implant for APBI using HDR multicatheter brachytherapy for Japanese patients with breast cancer and their cosmetic outcomes. Journal of Clinical Oncology. 2011;29(15). PubMed PMID: WOS:000208880303153.

325. Schlacher K, Christ N, Siaud N, Egashira A, Wu H, Jasin M. Double-strand break repair-independent role for BRCA2 in blocking stalled replication fork degradation by MRE11. Cell. 2011;145(4):529-42.

326. Senkesen, Kucucuk H, Aslay IS, Tezcanli E, Goksel E, Garipagaoglu M, et al. Dosimetric comparison of external radiotherapy techniques and high dose rate brachytherapy for breast cancer boost. Radiotherapy and Oncology. 2011;99:S252.

327. Shackleford TJ, Zhang QX, Tian L, Vu TT, Korapati AL, Baumgartner AM, et al. Stat3 and CCAAT/enhancer binding protein beta (C/EBP-beta) regulate Jab1/CSN5 expression in mammary carcinoma cells. Breast Cancer Research. 2011;13(3). doi: 10.1186/bcr2902. PubMed PMID: WOS:000295797100017.

328. Shaikhibrahim Z, Lindstrot A, Buettner R, Wernert N. Analysis of laser-microdissected prostate cancer tissues reveals potential tumor markers. International journal of molecular medicine. 2011;28(4):605-11. doi: 10.3892/ijmm.2011.746. PubMed PMID: WOS:000294532900019.

329. Skowronek J, Chichel A, Kanikowski M, Zwierzchowski G, Burchardt W. Pulsed dose rate brachytherapy as the boost after external beam radiation therapy and breast-conserving surgery of T1-T2 breast cancer. Radiotherapy and Oncology. 2011;99:S255.

330. Sun Z, Asmann YW, Kalari KR, Bot B, Eckel-Passow JE, Baker TR, et al. Deep sequence analysis of the relationship between gene expression, CpG island methylation, and gene copy number in breast cancer cells. Cancer research. 2011;71(8 SUPPL. 1):no pagination.

331. Tokunaga E, Yuichi H, Akiyoshi S, Okada S, Yamashita N, Morita M, et al. The expression of GATA-3 and FOXA1 in breast cancer: The biomarkers of hormonal sensitivity in luminal type tumors. Annals of Oncology. 2011;22:ix35.

332. Wallace M, Shah C, Mitchell C, John W, Vicini F. Twelve year clinical outcomes with interstitial accelerated partial breast irradiation. Radiotherapy and Oncology. 2011;99:S29.

333. Banerji S, Cibulskis K, Rangel-Escareno C, Brown KK, Carter SL, Frederick AM, et al. Sequence analysis of mutations and translocations across breast cancer subtypes. Nature. 2012;486(7403):405-9. doi: 10.1038/nature11154. PubMed PMID: WOS:000305466800044.

334. Bluekens AMJ, Holland R, Karssemeijer N, Broeders MJM, Den Heeten GJ. Comparison of digital screening mammography and screen-film mammography in the early detection of clinically relevant cancers: A multicenter study. Radiology. 2012;265(3):707-14.

335. Ellis MJ, Ding L, Shen D, Luo JQ, Suman VJ, Wallis JW, et al. Whole-genome analysis informs breast cancer response to aromatase inhibition. Nature. 2012;486(7403):353-60. doi: 10.1038/nature11143. PubMed PMID: WOS:000305466800034.

336. Miyamoto H, Izumi K, Yao JL, Li Y, Yang Q, McMahon LA, et al. GATA binding protein 3 is down-regulated in bladder cancer yet strong expression is an independent predictor of poor prognosis in invasive tumor. Human pathology. 2012;43(11):2033-40. doi: 10.1016/j.humpath.2012.02.011. PubMed PMID: WOS:000310654500029.

337. Perez AR, Carrizosa MCL, Ots PMS, Gomez J, Ortuno JZ, Garrido JDS, et al. Conservative surgery, external radiotherapy, and HDR brachytherapy in a single fraction of 7 Gy in early breast cancer: long-term toxicity and esthetic assessment. Clinical & Translational Oncology. 2012;14(12):953-60. doi: 10.1007/s12094-012-0881-4. PubMed PMID: WOS:000312125600011.

338. Perez AR, Ots PMS, Carrizosa MCL, Gomez J, Ortuno JZ, Garrido JDS, et al. Early-stage breast cancer conservative treatment: high-dose-rate brachytherapy boost in a single fraction of 700 cGy to the tumour bed. Clinical & Translational Oncology. 2012;14(5):362-8. doi: 10.1007/s12094-012-0809-z. PubMed PMID: WOS:000303678700008.

339. Polgar C, Major T, Sulyok Z, Frohlich G, Szabo E, Savolt A, et al. [Second breast-conserving surgery and reirradiation with interstitial high-dose-rate brachytherapy for the management of intra-breast recurrences -- 5-year results]. Magyar onkologia. 2012;56(2):68-74.

340. Ribeiro HB, Ergonenc HY, Demirel C, Buhler H, Adamietz IA, Galalae R. Short-term outcome of breast-conserving surgery (BCS) and mammosite applicator based interstitial high-dose rate brachytherapy (HDR-BT) in women with breast cancer (BC): preliminary results from a two-institution collaboration. Strahlentherapie Und Onkologie. 2012;188(8):739-. PubMed PMID: WOS:000306595500063.

341. Rodriguez Perez A, Lopez Carrizosa MC, Samper Ots PM, Perez-Regadera Gomez JF, Zapatero Ortuno J, Saez Garrido JDD, et al. Conservative surgery, external radiotherapy, and HDR brachytherapy in a single fraction of 7 Gy in early breast cancer: Long-term toxicity and esthetic assessment. Clinical and Translational Oncology. 2012;14(12):953-60.

342. Rodriguez Perez A, Samper Ots PM, Lopez Carrizosa MC, Perez-Regadera Gomez JF, Zapatero Ortuno J, Saez Garrido Jde D, et al. Early-stage breast cancer conservative treatment: high-dose-rate brachytherapy boost in a single fraction of 700 cGy to the tumour bed. Clinical & translational oncology : official publication of the Federation of Spanish Oncology Societies and of the National Cancer Institute of Mexico. 2012;14(5):362-8. Epub 2012/05/04. PubMed PMID: 22551542.

343. Seo MJ, Liu X, Chang M, Park JH. GATA-binding protein 1 is a novel transcription regulator of peroxiredoxin 5 in human breast cancer cells. International journal of oncology. 2012;40(3):655-64. Epub 2011/10/25. doi: 10.3892/ijo.2011.1236. PubMed PMID: 22020876.

344. Bockhorn J, Dalton R, Nwachukwu C, Huang SM, Prat A, Yee K, et al. MicroRNA-30c inhibits human breast tumour chemotherapy resistance by regulating TWF1 and IL-11. Nature communications. 2013;4. doi: 10.1038/ncomms2393. PubMed PMID: WOS:000316614600063.

345. Buchwalter G, Hickey MM, Cromer A, Selfors LM, Gunawardane RN, Frishman J, et al. PDEF Promotes Luminal Differentiation and Acts as a Survival Factor for ER-Positive Breast Cancer Cells. Cancer cell. 2013;23(6):753-67. doi: 10.1016/j.ccr.2013.04.026. PubMed PMID: WOS:000320350900009.

346. Calvo J, Sanchez-Cid L, Munoz M, Lozano JJ, Thomson TM, Fernandez PL. Infrequent Loss of Luminal Differentiation in Ductal Breast Cancer Metastasis. PloS one. 2013;8(10). doi: 10.1371/journal.pone.0078097. PubMed PMID: WOS:000326032600071.

347. Garsa AA, Ferraro DJ, DeWees T, Margenthaler JA, Naughton M, Aft R, et al. Analysis of fat necrosis after adjuvant high-dose-rate interstitial brachytherapy for early stage breast cancer. Brachytherapy. 2013;12(2):99-106.

348. Garsa AA, Ferraro DJ, Dewees TA, Deshields TL, Margenthaler JA, Cyr AE, et al. A prospective longitudinal clinical trial evaluating quality of life after breast-conserving surgery and high-dose-rate interstitial brachytherapy for early-stage breast cancer. International Journal of Radiation Oncology Biology Physics. 2013;87(5):1043-50.

349. Harmon JF, Rice BK. Comparison of planning techniques when air/fluid is present using the strut-adjusted volume implant (SAVI) for HDR-based accelerated partial breast irradiation. Journal of Applied Clinical Medical Physics. 2013;14(6):264-73. PubMed PMID: WOS:000326931100020.

350. Kobayashi T, Iwaya K, Moriya T, Yamasaki T, Tsuda H, Yamamoto J, et al. A simple immunohistochemical panel comprising 2 conventional markers, Ki67 and p53, is a powerful tool for predicting patient outcome in luminal-type breast cancer. BMC clinical pathology. 2013;13(1):no pagination.

351. Kocak ME, Barisik NO, Mayadagli A, Gemici C, Eren M, Naki M, et al. The Prognostic and Predictive Value of Expression of P-AKT in Patients with FIGO IIB Locally Advanced Cervical Cancer under Chemotherapy. Uhod-Uluslararasi Hematoloji-Onkoloji Dergisi. 2013;23(3):153-9. doi: 10.4999/uhod.11070. PubMed PMID: WOS:000325121400002.

352. Lee EK, Yuan F, Templeton A, Yao R, Kiel K, Chu JCH. Biological Planning for High-Dose-Rate Brachytherapy: Application to Cervical Cancer Treatment. Interfaces. 2013;43(5):462-76. doi: 10.1287/inte.2013.0711. PubMed PMID: WOS:000326432000006.

353. Roy S, Devleena, Maji T, Chaudhuri P, Lahiri D, Biswas J. Tumor bed boost in breast cancer: Brachytherapy versus electron beam. Indian Journal of Medical and Paediatric Oncology. 2013;34(4):257-63.

354. Bockhorn J, Prat A, Chang YF, Liu X, Huang SM, Shang M, et al. Differentiation and Loss of Malignant Character of Spontaneous Pulmonary Metastases in Patient-Derived Breast Cancer Models. Cancer research. 2014;74(24):7406-17. doi: 10.1158/0008-5472.CAN-14-1188. PubMed PMID: WOS:000346363900025.

355. Chandiramani N, Kenny PA. Comparative analysis of GATA3 mutation profiles between Asian and Western patients with breast cancer: Is there really a difference? Cancer. 2014;120(17):2778-9.

356. Chen PY, Pietron A, Wallace M, Ye H, Wobb J, Dekhne N, et al. The evolution of accelerated partial breast irradiation: Comparative analysis of clinical outcomes with interstitial vs balloon-based treatment. Brachytherapy. 2014;13:S17.

357. de la Puente P, Azab AK. Delivery systems for brachytherapy. Journal of controlled release : official journal of the Controlled Release Society. 2014;192:19-28.

358. Heim D, Budczies J, Stenzinger A, Treue D, Hufnagl P, Denkert C, et al. Cancer beyond organ and tissue specificity: Next-generation-sequencing gene mutation data reveal complex genetic similarities across major cancers. International Journal of Cancer. 2014;135(10):2362-9.

359. Huang Y, Ma M, Chen SW, Dai J, Chen F, Wang ZZ. Construction of multifunctional organic-inorganic hybrid Bi2S3-PLGA capsules for highly efficient ultrasound-guided radiosensitization of brachytherapy. Rsc Advances. 2014;4(51):26861-5. doi: 10.1039/c4ra02785j. PubMed PMID: WOS:000338640200038.

360. Keshari RP, Wang W, Zhang Y, Wang DD, Li YF, Yuan SQ, et al. Decreased Expression of the GATA3 Gene Is Associated with Poor Prognosis in Primary Gastric Adenocarcinoma. PloS one. 2014;9(2). doi: 10.1371/journal.pone.0087195. PubMed PMID: WOS:000330631800021.

361. Moskal A, Pisa PT, Ferrari P, Byrnes G, Freisling H, Boutron-Ruault MC, et al. Nutrient Patterns and Their Food Sources in an International Study Setting: Report from the EPIC Study. PloS one. 2014;9(6). doi: 10.1371/journal.pone.0098647. PubMed PMID: WOS:000336841400042.

362. Nguewa P, Manrique I, Diaz R, Redrado M, Parrondo R, Perez-Stable C, et al. Id-1B, an Alternatively Spliced Isoform of the Inhibitor of Differentiation-1, Impairs Cancer Cell Malignancy Through Inhibition of Proliferation and Angiogenesis. Current molecular medicine. 2014;14(1):151-62. PubMed PMID: WOS:000329187800012.

363. Nicholas Lukens J, Gamez M, Hu K, Harrison LB. Modern brachytherapy. Seminars in oncology. 2014;41(6):831-47.

364. Rodrigues M, Rodini CDO, Xavier F, Paiva KB, Severino P, Moyses RA, et al. PROX1 Gene is Differentially Expressed in Oral Cancer and Reduces Cellular Proliferation. Medicine. 2014;93(28). doi: 10.1097/MD.0000000000000192. PubMed PMID: WOS:000346762200005.

365. Rosell M, Nevedomskaya E, Stelloo S, Nautiyal J, Poliandri A, Steel JH, et al. Complex Formation and Function of Estrogen Receptor alpha in Transcription Requires RIP140. Cancer research. 2014;74(19):5469-79. doi: 10.1158/0008-5472.CAN-13-3429. PubMed PMID: WOS:000343118900013.

366. Trombetta M, Hall M, Julian TB. Long-term followup of breast preservation by re-excision and balloon brachytherapy after ipsilateral breast tumor recurrence. Brachytherapy. 2014;13(5):488-92. doi: 10.1016/j.brachy.2014.05.017. PubMed PMID: WOS:000341068200008.

367. Wheler JJ, Parker BA, Lee JJ, Atkins JT, Janku F, Tsimberidou AM, et al. Unique molecular signatures as a hallmark of patients with metastatic breast cancer: Implications for current treatment paradigms. Oncotarget. 2014;5(9):2349-54. PubMed PMID: WOS:000336966600002.

368. Xia HJ, He BL, Wang CY, Zhang HL, Ge GZ, Zhang YX, et al. PTEN/PIK3CA genes are frequently mutated in spontaneous and medroxyprogesterone acetate-accelerated 7,12-dimethylbenz(a)anthracene-induced mammary tumours of tree shrews. European Journal of Cancer. 2014;50(18):3230-42.

369. Yang YA, Ahn YH, Chen YL, Tan XC, Guo L, Gibbons DL, et al. ZEB1 sensitizes lung adenocarcinoma to metastasis suppression by PI3K antagonism. Journal of Clinical Investigation. 2014;124(6):2696-708. doi: 10.1172/JCI72171. PubMed PMID: WOS:000336868800040.

370. Zarva A, Mohnike K, Damm R, Ruf J, Seidensticker R, Ulrich G, et al. Safety of Repeated Radioembolizations in Patients with Advanced Primary and Secondary Liver Tumors and Progressive Disease After First Selective Internal Radiotherapy. Journal of Nuclear Medicine. 2014;55(3):360-6. doi: 10.2967/jnumed.113.127662. PubMed PMID: WOS:000332352100014.

371. Zuo W, Yu KD, Shao ZM. Reply to comparative analysis of GATA3 mutation profiles between Asian and Western patients with breast cancer: Is there really a difference? Cancer. 2014;120(17):2779-80.

372. Azim HA, Nguyen B, Brohee S, Zoppoli G, Sotiriou C. Genomic aberrations in young and elderly breast cancer patients. BMC medicine. 2015;13. doi: 10.1186/s12916-015-0504-3. PubMed PMID: WOS:000363137900001.

373. Banet N, Gown AM, Shih Ie M, Kay Li Q, Roden RB, Nucci MR, et al. GATA-3 expression in trophoblastic tissues: an immunohistochemical study of 445 cases, including diagnostic utility. The American journal of surgical pathology. 2015;39(1):101-8. Epub 2014/09/05. doi: 10.1097/pas.0000000000000315. PubMed PMID: 25188865; PubMed Central PMCID: PMCPMC4268033.

374. Gong C, Fujino K, Monteiro LJ, Gomes AR, Drost R, Davidson-Smith H, et al. FOXA1 repression is associated with loss of BRCA1 and increased promoter methylation and chromatin silencing in breast cancer. Oncogene. 2015;34(39):5012-24. doi: 10.1038/onc.2014.421. PubMed PMID: WOS:000361693300002.

375. Hoang LL, Tacha D, Bremer RE, Haas TS, Cheng L. Uroplakin II (UPII), GATA3, and p40 are Highly Sensitive Markers for the Differential Diagnosis of Invasive Urothelial Carcinoma. Applied Immunohistochemistry & Molecular Morphology. 2015;23(10):711-6. doi: 10.1097/PAI.0000000000000143. PubMed PMID: WOS:000365277000006.

376. Li RH, Campos J, Iida J. A Gene Regulatory Program in Human Breast Cancer. Genetics. 2015;201(4):1341-+. doi: 10.1534/genetics.115.180125. PubMed PMID: WOS:000366386500006.

377. Paryani NN, Vallow L, Magalhaes W, Heckman MG, Kim S, Smith A, et al. The incidence of fat necrosis in balloon-based breast brachytherapy. Journal of Contemporary Brachytherapy. 2015;7(1):29-34.

378. Si W, Huang W, Zheng Y, Yang Y, Liu X, Shan L, et al. Dysfunction of the Reciprocal Feedback Loop between GATA3- and ZEB2-Nucleated Repression Programs Contributes to Breast Cancer Metastasis. Cancer cell. 2015;27(6):822-36.

379. Teo ZL, Loi S. Molecular segmentation of luminal breast: Reality in 2015? Breast (Edinburgh, Scotland). 2015;24:S41-S3.

380. Urano M, Nagao T, Miyabe S, Ishibashi K, Higuchi K, Kuroda M. Characterization of mammary analogue secretory carcinoma of the salivary gland: discrimination from its mimics by the presence of the ETV6-NTRK3 translocation and novel surrogate markers. Human pathology. 2015;46(1):94-103. Epub 2014/12/03. doi: 10.1016/j.humpath.2014.09.012. PubMed PMID: 25456394.

381. Ashaie MA, Chowdhury EH. Cadherins: The superfamily critically involved in breast cancer. Current pharmaceutical design. 2016;22(5):616-38.

382. Aumsuwan P, Khan SI, Khan IA, Ali Z, Avula B, Walker LA, et al. The anticancer potential of steroidal saponin, dioscin, isolated from wild yam (Dioscorea villosa) root extract in invasive human breast cancer cell line MDA-MB-231 in vitro. Archives of biochemistry and biophysics. 2016;591:98-110.

383. Bao Y, Wang AT, Mo JF. S100A8/A9 is associated with estrogen receptor loss in breast cancer. Oncology letters. 2016;11(3):1936-42. doi: 10.3892/ol.2016.4134. PubMed PMID: WOS:000371634000053.

384. Dieci MV, Smutna V, Scott V, Yin GL, Xu R, Vielh P, et al. Whole exome sequencing of rare aggressive breast cancer histologies. Breast cancer research and treatment. 2016;156(1):21-32. doi: 10.1007/s10549-016-3718-y. PubMed PMID: WOS:000372258800003.

385. El Hag MI, Ha J, Farag R, El Hag AM, Michael CW. Utility of GATA-3 in the work-Up of breast adenocarcinoma and its differential diagnosis in serous effusions:: A Cell-Block Microarray Study. Diagnostic cytopathology. 2016. Epub 2016/06/25. doi: 10.1002/dc.23521. PubMed PMID: 27338760.

386. Haraguchi T, Miyoshi H, Hiraoka K, Yokoyama S, Ishibashi Y, Hashiguchi T, et al. GATA3 Expression Is a Poor Prognostic Factor in Soft Tissue Sarcomas. PloS one. 2016;11(6). doi: 10.1371/journal.pone.0156524. PubMed PMID: WOS:000377184700049.

387. Pichler R, Fritz J, Zavadil C, Schafer G, Culig Z, Brunner A. Tumor-infiltrating immune cell subpopulations influence the oncologic outcome after intravesical bacillus calmette-guerin therapy in bladder cancer. Oncotarget. 2016;7(26):39916-30. doi: 10.18632/oncotarget.9537. PubMed PMID: WOS:000378614700075.

388. Xie W, Qin W, Qin AP, Zhao ZH, Wang K, Kang YL, et al. GATA1 promotes tumorigenesis and metastasis in breast cancer by cooperating with ZEB2. International journal of clinical and experimental pathology. 2016;9(4):4167-78. PubMed PMID: WOS:000375409600002.

389. Engelsen IB, Stefansson IM, Akslen LA, Salvesen HB. GATA3 expression in estrogen receptor alpha-negative endometrial carcinomas identifies aggressive tumors with high proliferation and poor patient survival. American journal of obstetrics and gynecology. 2008;199(5). doi: 10.1016/j.ajog.2008.04.043. PubMed PMID: WOS:000260585800037.

390. Bong PN, Zakaria Z, Muhammad R, Abdullah N, Ibrahim N, Emran NA, et al. Expression and mutational analysis of GATA3 in Malaysian breast carcinomas. The Malaysian journal of pathology. 2010;32(2):117-22. Epub 2011/02/19. PubMed PMID: 21329183.

391. Wu YY, Elshimali Y, Sarkissyan M, Mohamed H, Clayton S, Vadgama JV. Expression of FOXO1 is associated with GATA3 and Annexin-1 and predicts disease-free survival in breast cancer. American journal of cancer research. 2012;2(1):104-15. PubMed PMID: WOS:000208854500008.

392. Cimino-Mathews A, Subhawong AP, Illei PB, Sharma R, Halushka MK, Vang R, et al. GATA3 expression in breast carcinoma: utility in triple-negative, sarcomatoid, and metastatic carcinomas. Human pathology. 2013;44(7):1341-9. doi: 10.1016/j.humpath.2012.11.003. PubMed PMID: WOS:000321225000016.

393. Braxton DR, Cohen C, Siddiqui MT. Utility of GATA3 immunohistochemistry for diagnosis of metastatic breast carcinoma in cytology specimens. Diagnostic cytopathology. 2015;43(4):271-7. Epub 2014/08/05. doi: 10.1002/dc.23206. PubMed PMID: 25088841.

394. Dang DN, Raj G, Sarode V, Molberg KH, Vadlamudi RK, Peng Y. Significantly increased PELP1 protein expression in primary and metastatic triple-negative breast carcinoma: comparison with GATA3 expression and PELP1's potential role in triple-negative breast carcinoma. Human pathology. 2015;46(12):1829-35. doi: 10.1016/j.humpath.2015.07.023. PubMed PMID: WOS:000365836600006.

395. Lew M, Pang JC, Jing X, Fields KL, Roh MH. Young investigator challenge: The utility of GATA3 immunohistochemistry in the evaluation of metastatic breast carcinomas in malignant effusions. Cancer Cytopathol. 2015;123(10):576-81. Epub 2015/10/16. doi: 10.1002/cncy.21574. PubMed PMID: 26465236.

396. Ni YB, Tsang JY, Chan SK, Tse GM. GATA-binding protein 3, gross cystic disease fluid protein-15 and mammaglobin have distinct prognostic implications in different invasive breast carcinoma subgroups. Histopathology. 2015;67(1):96-105. Epub 2014/11/27. doi: 10.1111/his.12625. PubMed PMID: 25425335.

397. Ping Z, Xia YC, Shen TS, Parekh V, Siegal GP, Eltoum IE, et al. A microscopic landscape of the invasive breast cancer genome. Scientific reports. 2016;6. doi: 10.1038/srep27545. PubMed PMID: WOS:000377688900001.

398. Gonzalez RS, Wang J, Kraus T, Sullivan H, Adams AL, Cohen C. GATA-3 expression in male and female breast cancers: comparison of clinicopathologic parameters and prognostic relevance. Human pathology. 2013;44(6):1065-70. Epub 2012/12/26. doi: 10.1016/j.humpath.2012.09.010. PubMed PMID: 23266442.

399. Liu J, Prager-van der Smissen WJ, Look MP, Sieuwerts AM, Smid M, Meijer-van Gelder ME, et al. GATA3 mRNA expression, but not mutation, associates with longer progression-free survival in ER-positive breast cancer patients treated with first-line tamoxifen for recurrent disease. Cancer letters. 2016;376(1):104-9. Epub 2016/03/29. doi: 10.1016/j.canlet.2016.03.038. PubMed PMID: 27018307.
